# Characterization of Calmodulin-Free Murine Inducible Nitric-Oxide Synthase

**DOI:** 10.1371/journal.pone.0121782

**Published:** 2015-03-30

**Authors:** Latika Nagpal, Koustubh Panda

**Affiliations:** Department of Biotechnology & Guha Center for Genetic Engineering & Biotechnology, University of Calcutta, Kolkata, India; Argonne National Laboratory, UNITED STATES

## Abstract

Nitric-Oxide Synthase (NOS), that produces the biological signal molecule Nitric-Oxide (NO), exists in three different isoforms called, neuronal (nNOS), endothelial (eNOS) and inducible (iNOS). All NOS isoforms require post-translational interaction with the calcium-binding protein, calmodulin (CaM) for manifesting their catalytic activity. However, CaM has been suggested to control the translational assembly of the enzyme as well, particularly in helping its inducible isoform, iNOS assume a stable, heme-replete, dimeric and active form. Expression of recombinant murine iNOS in *E*.*coli* in the absence of CaM has been previously shown to give extremely poor yield of the enzyme which was claimed to be absolutely heme-free, devoid of flavins, completely monomeric and catalytically inactive when compared to the heme-replete, active, dimeric iNOS, generated through co-expression with CaM. In contrast, we found that although iNOS expressed without CaM does produce significantly low amounts of the CaM-free enzyme, the iNOS thus produced, is not completely devoid of heme and is neither entirely monomeric nor absolutely bereft of catalytic activity as reported before. In fact, iNOS synthesized in the absence of CaM undergoes compromised heme incorporation resulting in extremely poor dimerization and activity compared to its counterpart co-expressed with CaM. Moreover, such CaM-free iNOS has similar flavin content and reductase activity as iNOS co-expressed with CaM, suggesting that CaM may not be as much required for the functional assembly of the iNOS reductase domain as its oxygenase domain. LC-MS/MS-based peptide mapping of the CaM-free iNOS confirmed that it had the same full-length sequence as the CaM-replete iNOS. Isothermal calorimetric measurements also revealed high affinity for CaM binding in the CaM-free iNOS and thus the possible presence of a CaM-binding domain. Thus CaM is essential but not indispensible for the assembly of iNOS and such CaM-free iNOS may help in elucidating the role of CaM on iNOS catalysis.

## Introduction

Nitric-Oxide Synthase (NOS) that produces the physiologically versatile free radical, Nitric-oxide (NO) in our body is active only in its homodimeric form and catalyzes the NADPH-dependent oxidation of its substrate, L-Arginine (L-Arg) to its biologically active product, NO along with citrulline. The enzyme is comprised of a C-terminal flavoprotein rich reductase domain, that binds NADPH, FAD and FMN, a central calmodulin (CaM) binding motif and a N-terminal domain, known as the oxygenase domain comprising of the heme and redox cofactor, (R)-5,6,7,8-tetrahydrobiopterin (H_4_B) binding sites as well as the binding site for the substrate, L-Arg [1]. The NOS enzyme exists in three different isoforms—endothelial (eNOS), neuronal (nNOS) and inducible (iNOS), which despite sharing significant sequence homology are differentially regulated based on their cellular localizations [1–2]. All NOS isoforms contain FAD, FMN, NADPH, heme and H_4_B binding sites besides the substrate and CaM-binding sites. CaM binds and activates the Ca^2+^-dependent constitutive NOS (cNOS) isoforms, eNOS and nNOS apparently at elevated cellular Ca^2+^ concentrations whereas, iNOS primarily remains irreversibly bound to CaM in a Ca^2+^-independent manner [3–4]. In fact, iNOS is usually not expressed during routine physiological events, but its expression is typically induced by pathological stimuli like bacterial lipopolysaccharide, cytokines and exposure to various inflammatory xenobiotics. Although primarily identified in macrophages, expression of the enzyme can be stimulated in virtually any cell or tissue, provided that the appropriate agents get deployed for inducing its synthesis. Once expressed with CaM, iNOS is catalytically active and is not regulated by intracellular Ca^2+^ concentrations [5–6].

The constitutive NOSs, eNOS and nNOS are mostly involved in the synthesis of physiologically optimal levels (nM levels) of NO and respectively help sustain specific physiological functions in the endothelial cells and brain or nerve cells [7]. In contrast, iNOS which has almost six-fold and ten-fold native catalytic activity of that of nNOS and eNOS [8] respectively, is usually involved in the production of relatively high levels of NO (μM levels) when stimulated by various intracellular or extracellular inflammatory stimuli, primarily under pathological or disease conditions [9].

The full-length murine iNOS protein (iNOSfl) consists of 1144 amino acids with a molecular weight of around 130 KDa of which the N-terminal oxygenase and the C-terminal reductase domains are defined by amino acids from 1–498 and 631–1144 respectively with the intervening CaM binding domain composed of about 24 amino acids (505–528) [10]. It has been proposed that CaM binding to iNOSfl helps in mobilizing trans electron transfer between the reductase domain of one iNOS subunit and the heme of the oxygenase domain of the adjacent subunit in a iNOSfl dimer. The typical NOS dimer is formed through the association of the oxygenase domains of two monomeric NOS subunits with their reductase domains remaining as freely hanging tails in their CaM unbound state. It is also suggested that CaM binding to CaM-free NOS apparently mobilizes the contact of the reductase domains with the opposite oxygenase domains in the NOS dimer through ‘domain swapping’ leading to electron transfer to the heme and oxidation of the substrate, L-Arg to citrulline and the biologically active NO. This apparently justifies why NOS is only active in its CaM-bound dimeric form [3,11].

Earlier reports on CaM-free full-length iNOS (iNOSfl) suggest that CaM must be co-expressed with iNOS for inducing high level expression of the protein. Such CaM-bound iNOS, besides being predominantly dimeric contains normal quantities of heme and flavins and also has high NO synthesis activity. In contrast, iNOS expressed in the absence of CaM was found to be monomeric with no bound heme and flavins and lacked NO synthesis activity [12].

However, as the CaM-free full-length iNOS has a very poor yield compared to the CaM co-expressed form of the iNOSfl protein [12], we expressed and purified this protein from a bacterial culture expressing the recombinant protein using almost six times the culture volume used for purification of the wild-type CaM-replete iNOSfl protein. The CaM-free iNOSfl protein, thus purified, was detected through immunoblotting using iNOS monoclonal antibody against confirming molecular weight markers and also analyzed through LC-MS/MS based peptide mapping to confirm its structural integrity and composition.

Although, interaction studies of CaM with synthesized peptides representing the CaM binding motifs of the three NOS isoforms [13–14] or combination of the two constitutive isoforms of NOS (nNOS and eNOS) [15] that apparently do not require CaM for their translational assembly, have been carried out before, no study has conjointly examined the comparative CaM binding affinities of all the three NOS isoforms and their possible consequence on their relative catalytic activities. This has also left open the question of the physiological relevance of such binding studies on synthetic NOS CaM binding peptides with CaM, whose data significantly differ from that obtained with the wild-type NOSfl protein counterparts [15]. More importantly, such CaM peptide-binding data comes of little use for correlating the possible role of CaM binding on the structure-function relationship governing the differential catalytic behaviors of the three NOS isoforms both at the translational and post-translational levels. Thus our successful characterization of the CaM-free iNOSfl protein and demonstration of its ability to exist without any pre-association with CaM as well as its capability to manifest humble NO synthesis activity after associating with CaM, creates the much needed premises for conjointly studying the comparative role of CaM binding on the catalytic properties of all the three NOS isoforms in the future.

## Materials and Methods

### Chemicals

Resins used for purification of the iNOS proteins were procured from GE Healthcare, USA while all other reagents and chemicals used were obtained either from Sigma-Aldrich, USA or Merck, Germany and were of analytical grade.

### Bacterial transformation

The TransformAid Bacterial Transformation Kit (Fermentas, USA) was used to transform *Escherichia coli* (*E*.*coli*) strains BL21(DE3) and DH5α with iNOS and CaM plasmid DNAs either separately or together using the expression vector pCW through standard procedures [12]. The transformed bacteria were then grown overnight in cultures and stored at -80°C as glycerol stocks for further use.

### Plasmid DNA isolation and detection

All transformed cultures were grown overnight and the plasmid DNAs were isolated using the QIAprep Spin Miniprep kit (Qiagen) as per the protocol provided by the manufacturer. The plasmid DNA isolated were stored at -20°C in Tris-EDTA buffer or deionized water. The isolated DNA samples were loaded in 0.8% agarose gel containing ethidium bromide (final concentration—0.2 μg/ml; Stock solution—10 mg/ml) and the DNA bands in the agarose gel visualized using a UV- trans-illuminator.

### Purification of iNOSfl proteins co-expressed with and without CaM in bacteria

iNOSfl protein co-expressed with CaM was purified from a 72 h room temperature culture of 4 litre Terrific broth supplemented with 50 μg/ml ampicillin, 50 μg/ml chloramphenicol and 4 ml (10%) glycerol and pre-inoculated with 400 ml of overnight culture of BL21(DE3) containing both the iNOSfl and CaM expressing plasmids. Protein expression was induced when absorbance reached 0.8–1.0 at 600 nm by addition of 1 mM IPTG along with a heme precursor, δ-Ala (delta-aminolevulinic acid), to a final concentration of 400 μM and incubated for another 72 h at room temperature under aerobic environment (25°C). Cell pellets were then harvested by centrifugation and resuspended in 1/5 culture volume of lysis buffer (40 mM EPPS, pH 7.6, DTT 3mM) in the presence of L-Arg (1 mM) and H_4_B (10 μM). The bacterial cell debris was removed by centrifugation at 17,000 rpm for 30 min. The supernatant, containing the iNOSfl protein, was subjected to ammonium sulfate precipitation and was further precipitated by centrifugation. The supernatant was then loaded into a Ni-NTA resin (25 ml, GE Healthcare) column pre-charged with 50 mM NiSO_4_ and equilibrated with an equilibration buffer consisting of 40 mM EPPS buffer, pH 7.6, 10% glycerol, 1 mM PMSF/EtOH, 1mM L-Arg, 10 μM H_4_B and 250 mM NaCl. The column was then thoroughly washed with the EPPS buffer to remove all non-specifically bound proteins following which the bound iNOSfl protein was eluted with 160 mM imidazole. The Ni-NTA eluate was thereafter subjected to 2',5' ADP-Sepharose (GE Healthcare) column chromatography during which the column was equilibrated with 40 mM EPPS buffer (pH 7.4), 3 mM DTT, 2 μM FAD and 10% glycerol with 1 mM L-Arg and 10 μM H_4_B. The column was then washed thoroughly to remove all unbound proteins and the iNOS protein was finally eluted with 10 mM NADPH. The eluate containing the iNOSfl protein was concentrated using a 50-MWCO concentrator (Amicon) and the concentrated protein was dialyzed at 4°C against a dialysis buffer containing 40 mM EPPS (pH 7.6), 10% glycerol, 1 mM DTT, 250 mM NaCl along with 1 mM L-Arg and 10 μM H_4_B and thereafter stored in small aliquots at -85°C. The purity of the iNOSfl protein was checked spectrophotometrically and also through SDS-gel electrophoresis.

Bacterially expressed recombinant CaM-free full-length iNOS protein has a comparatively poor yield compared to the CaM co-expressed form of iNOSfl [12]. Recognizing this, the CaM-free iNOS protein was expressed and inoculated in a Terrific broth culture volume of almost six times higher culture volume (24 liters of Terrific broth culture) compared to that used for expressing the iNOSfl protein along with CaM expression. The protein was purified following similar protocols as mentioned above. The purity of the protein obtained was checked spectrophotometrically and also through SDS-PAGE analysis. The protein was also detected through Western blot using iNOS antibody and also by peptide mapping using Nano-LC-MS/MS based protein analysis.

### Gel filtration chromatography (FPLC)

Gel filtration chromatographic analysis of dimer-monomer content of iNOS proteins expressed in absence and presence of CaM was performed using ÄKTA purifier fast protein liquid chromatography system (GE Healthcare). Equal amounts of protein were loaded into a Superdex-200 gel filtration column (GE Healthcare) at 4°C. Chromatography was performed according to the protocol described previously [16]. Fractions of 500 μl were collected from size-exclusion column and part of it (100 μl) was subjected to 8% SDS-PAGE based immunoblotting of iNOSfl using mouse anti-iNOS monoclonal antibody. NO synthesis activity of each eluted fraction was also estimated using the Griess assay as described below.

### Low temperature SDS-PAGE of iNOS

Dimer and monomer contents of iNOS proteins were also examined using the low temperature SDS-PAGE method of Klatt *et al*. [17] with modifications. Equivalent amounts (in terms of protein content) of the CaM-replete and CaM-free iNOS was mixed with 20 μl of sample buffer containing 0.125 M Tris-HCl (pH 6.8), 2% (w/v) SDS, 20% (w/v) glycerol and 0.02% (w/v) bromophenol blue and subjected to SDS-PAGE in an ice-water bath at a constant current of 10 mA using a Mini Protean III apparatus (Bio-Rad). The casted gels and buffers used were all equilibrated at 4°C prior to the electrophoresis experiment to maintain the cold temperature.

### NO synthesis activity

NO synthesis of fractions (100 μl) collected from FPLC were assayed in a 96-microwell plates in an assay buffer containing 40 mM EPPS, 3 mM DTT, 4 μM FAD, 4 μM FMN, 10 μM H_4_B, 10 mM L-Arg, 0.5 mM EDTA, 1.2 mM CaCl_2_, 1 μM CaM, 0.1 mg/ml bovine serum albumin (BSA), 18 units/ml catalase and 10 units/ml superoxide dismutase. NADPH (10 mM) was added to trigger NO production at 37°C and the plate was incubated for 30 min before the reaction was frozen with the addition of 100 μl Griess reagent to each well. The absorbance of the red azo dye formed by the reaction of the Griess reagent and the nitrite produced as a result of NO solubilization in the aqueous reaction mixture was measured at 550 nm in a VersaMax microplate reader (Molecular Devices). The NO produced was evaluated using NaNO_2_ standards. Whereas, steady-state rates of NO synthesis were determined by the spectrophotometric oxyhemoglobin assay using a difference extinction coefficient of 38 mM^-1^ cm^-1^ for the oxyhemoglobin to methemoglobin transition at 401 nm [18]. The assay buffer used was the same as mentioned above except that 10 μM oxyhemoglobin and 0.5 μM NOS was used for the assay. The reactions were initiated by addition of 10 mM NADPH and the absorbance change at 401 nm was recorded at 25ºC to quantify the amount of NO produced.

### Spectrophotometric measurement of heme

UV-visible wavelength scans were recorded at room temperature on a Shimadzu UV-2450 spectrophotometer. For recording the spectra of CO binding to NOS, CO was first bubbled into the protein and sodium dithionite was further added and the proteins were subjected to wavelength scanning between 300 and 700 nm. The ferrous–CO adduct absorbance at 444 nm was used to determine heme content in the protein using an extinction coefficient of 74 mM^-1^ cm^-1^ (A_444_–A_500_)

### Heme content measurement using PIA-514

PIA-514, a highly sensitive and photostable iNOS heme-binding fluorescent compound developed by our group, was deployed to quantify the heme content in iNOS proteins expressed in the presence and absence of CaM. The protein was incubated with PIA-514 for 30 min to enable complete PIA-514 binding to the iNOS heme and subsequent estimation of heme-content by fluorescence imaging of the gel bands through excitation at a wavelength of 520 nm and emission at 560 nm.

### Immunoblotting for detection of iNOS and CaM proteins

Following electrophoresis, the proteins were transferred from the gels to PVDF membranes, blocked with 5% BSA and subjected to immunoblotting for iNOS using anti-mouse iNOS antibody (1:4000) (BD Transduction Laboratories) following standard protocols [19]. Protein bands representing the iNOSfl dimer and monomer were detected using mouse secondary antibody (1:10,000) and ECL chemiluminescence detection kit (GE Healthcare). Presence or absence of CaM association with the purified iNOSfl proteins expressed with or without CaM co-expression, was detected from SDS-PAGE protein immunoblots of equivalent amounts of the purified iNOSfl proteins using CaM-specific anti-mouse monoclonal antibody (1:100) (Sigma-Aldrich). Contents of the two iNOSfl enzymes in their purified protein preparations were determined using Western blot based densitometric evaluation of their immunoblot bands using anti-iNOSfl monoclonal antibodies (B.D Biosciences), prior to subjecting equivalent amounts of the two enzymes, determined therefrom, to separate CaM and iNOS immunoanalysis from replicate SDS-PAGE gels by Western blotting.

### In-solution trypsin digestion

iNOSfl proteins expressed and purified both in the presence and absence of CaM were diluted in a digestion buffer containing 50 mM ammonium bicarbonate solution. The disulfide bonds were reduced by adding freshly prepared 45 mM dithiothreitol (DTT) in 50 mM ammonium bicarbonate solution (1 nmol of protein was treated with 1μl of the DTT solution and incubated for 15 min at 50°C). For alkylation of cysteine residues, freshly prepared 100 mM solution of iodoacetamide (IAA) was dissolved in 50 mM ammonium bicarbonate solution (1 nmol of protein was alkylated by adding 1μl of the IAA solution and further incubated in the dark for 15 min at room temperature). Excess IAA in the protein sample was removed by addition of 1μl DTT solution. A small volume aliquot of the trypsin stock solution (1 mg/ml) was added to the above mix to obtain a final enzyme: substrate ratio of 1:50 (mass: mass). After addition of trypsin, the sample was incubated overnight at 37°C after which the enzyme activity was stopped by addition of a small aliquot of 10% trifluoroacetic acid (TFA).

### Liquid Chromatography-Mass Spectrometry Analysis (LC-MS/MS Analysis)

In-solution tryptic digested peptides were subjected to nano-LC-MS/MS analysis on a maXis impact mass spectrometer (Bruker Daltonics) equipped with a nano-advance LC (Bruker Daltonics, having a Biosphere C18 nano column (100 mm x 100 μm) (Nanoseparation, ) having C18 of 5 μm particle size and 120 Å pore size. The peptides were loaded onto the C18 trap column (5 μm, 120A, 10mm X 100 μm), Nano separation using an auto-sampler and desalted and concentrated for 2 min at a flow-rate of 5000 nL/min in isocratic mode. The mobile phase buffers used were—*Mobile Phase A*, consisting of 0.1% formic acid dissolved in MS grade water and *Mobile Phase B*, containing 0.1% formic acid dissolved in MS grade acetonitrile. Flow rate was automatically adjusted to 300 nl/min for gradient separation of desalted peptides using the following gradient protocol—5% buffer B to 40% buffer B was run for 30 min followed by 40% buffer B to 90% buffer B for 5 min; and 90% buffer B to 2% buffer B for 10 min. The total time for nano-LC-MS/ MS analysis was 52 minutes. The mass spectrometer was operated in a data-dependent fashion, the 20 most abundant precursor ions (threshold 3000 cts) selected from the MS spectrum were MS/MS fragmented via collision-induced dissociation (CID). Each full MS scan was acquired over a mass range of 50−2200 m/z. Dynamic exclusion was set with one repeating count (active exclusion after 3 spectra, released after 1 min). Flex analysis software version 3.4 was used to analyze the results of the peptide mapping.

### Isothermal Titration Calorimetry (ITC)

ITC experiment was performed for studying the thermodynamic binding interaction between the CaM-free iNOS and CaM and to confirm the presence as well as functional integrity of the CaM binding site in the CaM-free iNOS. The iNOSfl and CaM proteins were respectively dissolved in 200 μl and 2 ml ITC buffer (40 mM EPPS, 10% glycerol, 150 mM NaCl and 2 mM Ca^2+^) and the sample was degassed for 10 min before the titration. CaM binding rate to iNOSfl (-CaM) was determined by using 20 μM of the enzyme against 8 μM of CaM. Acquisition data was analyzed using MicroCal Origin Version 2.9 (MicroCal Software, Inc.). The heat of ligand dilution was determined by injecting ligand into the same ITC buffer and subtracted from the experimental values. The binding constants were quantified by fitting the evolved heat in single injection to a single-site binding model using a nonlinear least-squares method and the binding stoichiometry (n), the association constant (K_a_) and the enthalpy change (ΔH) were obtained from the fitted curve. The values of the Gibbs free energy change (ΔG) and the entropy change (ΔS) were calculated from the equation: ΔG = —RT ln Ka = ΔH—TΔS, where R is the gas constant and T is the absolute temperature in Kelvin.

### Cytochrome c and Ferricyanide reductase activity

The cytochrome c and ferricyanide reductase activities were determined by monitoring the change of absorbance at 550 nm and 420 nm, using extinction coefficients of 21 mM^-1^cm^-1^ and 1.01 mM^-1^cm^-1^ respectively as previously reported [20]. The assays were carried out in cuvettes at 25°C. The assay buffer contained 40 mM EPPS, pH 7.6, 4 μM FAD, 0.1 mg/ml BSA, 10 μg/ml CaM, 0.6 mM EDTA, 10 units /ml catalase, 10 units/ml superoxide dismutase to which either 0.1 mM cytochrome c or potassium ferricyanide was added. After the addition of the iNOSfl enzyme (50nM), the reaction was initiated by adding 0.1 mM NADPH and the rate of reduction of cytochrome c and ferricyanide determined from the slopes of the reaction curves.

### Measurement of flavin content of iNOSfl

To calculate the total flavin content in iNOS proteins, flavin fluorescence emission spectra were measured by exciting samples at 450 nm and flavin fluorescence emission was measured from 470 to 650 nm. Total flavin was calculated using a flavin (FMN and FAD) standard curve with the same measurement parameters. All spectra were corrected for instrumental artifacts by subtracting the baseline emission spectrum of the buffer.

### Measurement of apparent K_m_ values for L-Arg or H_4_B

Apparent K_m_ values were determined by fitting plots of the NO synthesis activity *versus* L-Arg or H_4_B concentration. In the case of K_m_ measurements for L-Arg, iNOSfl proteins were purified in the presence of L-Arg and H_4_B and thereafter dialysed against a buffer with a higher concentration of H_4_B (200 μM) and no L-Arg to remove L-Arg from the protein and obtain a H_4_B bound iNOSfl protein. Corresponding NO synthesis activities of the protein was determined using different concentration of L-Arg (1–20 mM) and plotted to determine K_m_ of L-Arg for iNOSfl. Similarly, for the K_m_ measurements for H_4_B, iNOSfl protein initially purified in the presence of L-Arg and H_4_B was dialysed against a buffer with a higher concentration of L-Arg (20 mM) and no H_4_B to obtain a H_4_B-free, L-Arg bound iNOSfl protein. Corresponding NO synthesis activities were plotted similarly against different concentrations of H_4_B (5–100 μM) to determine K_m_ of H_4_B for iNOSfl [21].

## Results and Discussion

### Expression and purification of recombinant murine iNOSfl protein in the absence and presence of CaM

**Fig 1:**
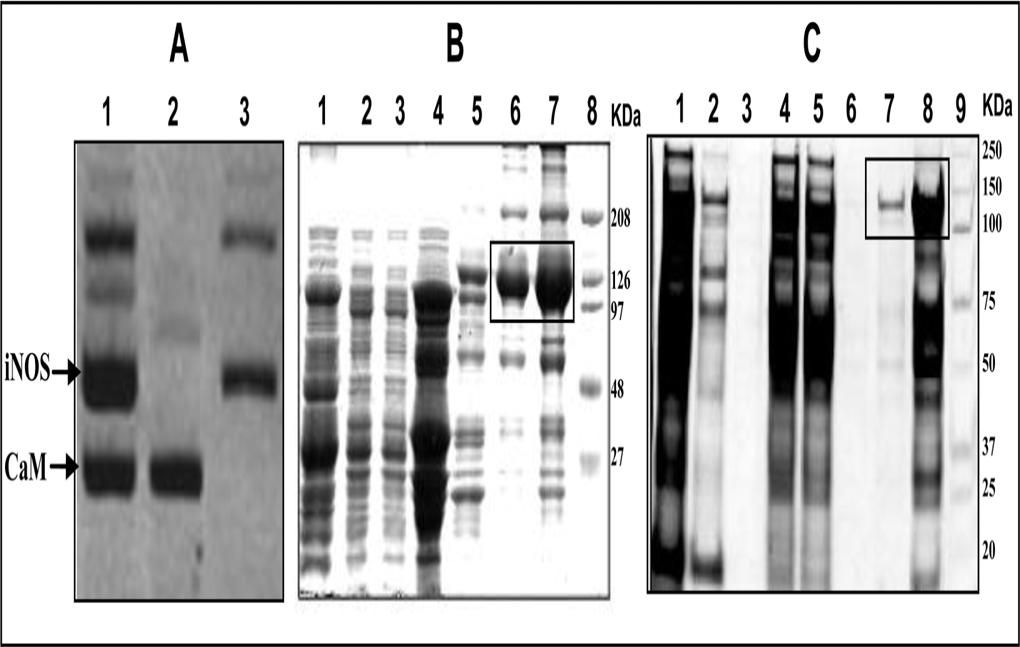
DNA and protein purification profiles of bacterially expressed recombinant iNOSfl in the presence and absence of CaM. **Panel A** shows the plasmid DNA profiles in BL21(DE3) *E*. *coli* cells containing iNOSfl expressing plasmids along with (lane 1) or without (lane 3) CaM expressing plasmids against only the CaM plasmid DNA (lane 2). 0.5 μg each of the isolated DNA samples were loaded in 0.8% agarose gel in which the DNA size ranged between 9.5 to 3.5 kb. **Panels B and C** show the 10% SDS-PAGE profiles of the protein fractions collected at each step of purification of iNOSfl co-expressed with CaM (B) and without CaM (C) respectively. SDS-PAGE protein profiles represent 10 μl of the indicated fractions after staining with Coomassie blue. **Panel B** depicts the protein profiles of fractions collected at different stages of purification of iNOSfl in the presence of CaM comprising of the suspension of the ammonium sulphate cut pellet of the bacterial lysate (Lane 1); Ni-NTA column flow through (Lane 2); Ni-NTA imidazole eluate (Lane 3); ADP column flow through (Lane 4); ADP column zero wash (Lane 5); ADP column NADPH eluate (Lane 6) and concentrated NADPH eluate of the purified iNOSfl protein expressed with CaM (Lane 7) along with standard molecular weight markers (Lane 8). Similarly, **Panel C** shows the protein profiles of fractions collected at different stages of purification of iNOSfl in the absence of CaM, namely the suspension of the ammonium sulphate cut pellet of the bacterial lysate (Lane 1); Ni-NTA column flow through (Lane 2); Ni column zero wash (Lane 3); Imidazole eluate (Lane 4); ADP column flow through (Lane 5); ADP column zero wash (Lane 6); ADP column NADPH eluate (Lane 7) and concentrated NADPH eluate of the purified iNOSfl (-CaM) protein (Lane 8) along with standard molecular weight markers (Lane 9). The pure iNOSfl bands are boxed in Panels B and C.

*E*.*coli* BL21(DE3) bacterial cells either transformed with murine iNOSfl expressing plasmids alone or co-transformed with CaM expressing plasmids were used for expressing iNOSfl proteins in the absence and presence of CaM. Fig 1, Panel A depicts the DNA profiles of the above transformed bacterial cells showing the successful incorporation of the iNOSfl expressing plasmids with (Fig 1A, Lane 1) and without the CaM expressing plasmid (Fig 1A, Lane 3) against the only CaM expressing plasmid as a reference (Fig 1A, Lane 2). Whereas Panels B and C show the SDS-PAGE protein profiles for the fractions defining the different purification stages of the iNOSfl proteins in the presence (Fig 1B) and absence of CaM (Fig 1C). Although the sequential Ni-NTA affinity column chromatography (utilizing its affinity for the His-6-tag at the N-terminal end of the recombinant iNOSfl protein) followed by the 2', 5', ADP-sepharose affinity chromatography (exploiting its affinity for the C-terminal NADPH binding domain) was carried out to ensure that only the full-length version of the iNOS protein (iNOSfl) consisting of the entire oxygenase and reductase domains is purified and not its fragmented or proteolyzed forms for final characterization, we did observe substantial amounts of proteins in the range of 50–75 kDa especially in case of the iNOSfl purified in the absence of CaM. This could be due to the fragmentation of the extremely fragile CaM-deficient iNOSfl protein post-purification and during electrophoretic separation besides contribution from bacterial proteins that manage to adhere to both the Ni-NTA as well as 2'-5' ADP resins. This perhaps also explains why we accomplished purification of about 100–120 mg of the purified iNOSfl protein from 1 litre of the bacterial culture when the enzyme was co-expressed with CaM in contrast to about 15–18 mg of the protein per litre bacterial culture when it was expressed in the absence of CaM. Such constraints compelled us to use almost six times more culture volume for the purification of the CaM-deficient enzyme compared to its CaM-replete counterpart for our comparative characterization studies.

### Relative characterization of the iNOSfl proteins expressed and purified in the presence and absence of CaM

**Fig 2:**
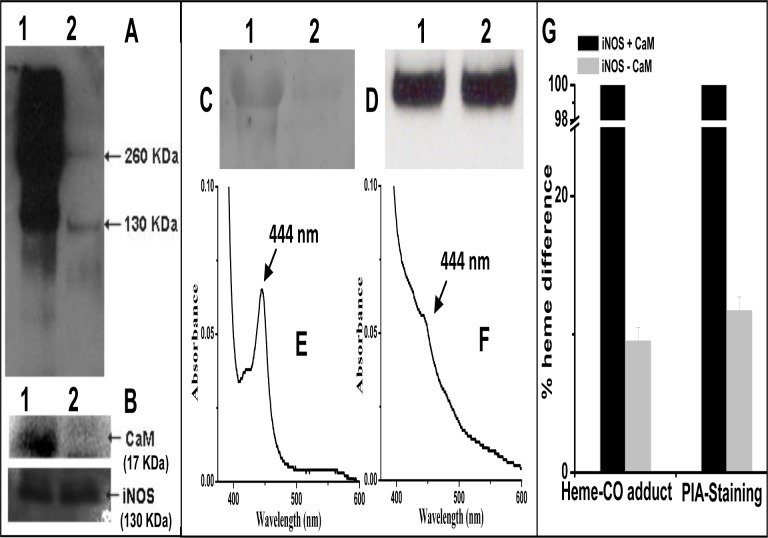
Immunoblot of purified iNOSfl co-expressed with and without CaM and PIA-514-based fluorescence SDS-PAGE for heme content estimation. **Panel A** shows the Western blot using anti-mouse iNOSfl monoclonal antibody for detection of possible NOS dimer and monomer in iNOSfl proteins (200 μg each) expressed with (Lane 1) and without CaM (Lane 2) through a low temperature SDS-PAGE (*see 'Materials and Methods'*). **Panel B** depicts the corresponding Western blot bands of CaM in a SDS-PAGE immunoblot of equivalent amounts of the purified iNOSfl proteins co-expressed with (Lane 1) and without (Lane 2) CaM expressing plasmids against their iNOSfl protein equivalence immunoblots. **Panel C** shows the fluorescence SDS-PAGE gel electrophoresis band images of heme in equivalent amounts of the iNOSfl proteins expressed in the presence (Lane 1) and absence (Lane 2) of CaM, based on Western blot based evaluation and analysis of equivalent amounts of the iNOSfl proteins (**Panel D**). The iNOSfl proteins were also pre-incubated with 2 μM of a newly developed iNOS-heme specific probe called PIA-514 (labeled with Alexafluor 514) and the resulting SDS-PAGE gel was scanned at an excitation of 520 nm and emission of 560 nm to visualize the heme-specific fluorescence bands shown in **Panel C**. **Panels E and F** show the corresponding levels of heme-CO peaks at 444 nm depicting relative heme content in the iNOSfl proteins expressed in the presence (**Panel E**) and absence (**Panel F**) of CaM. **Panel G** depicts the comparative percentage heme content of the CaM-deficient and CaM-replete iNOSfl proteins depicted in **Panels C** (PIA-514 staining) against that in **Panels E and F** (heme-CO-adduct estimation) considering heme content of the iNOSfl protein purified in the presence of CaM as 100%. Data represent three independent experiments done under similar conditions.

In order to determine whether co-expression with CaM is mandatory for making iNOS capable of dimerization, an essential post-translational modification required for rendering the enzyme active, we attempted to examine possible levels of dimer and monomer in the CaM-deficient iNOSfl protein against its CaM-replete counterpart through a previously standardized low-temperature SDS-PAGE based immunoblotting of iNOSfl [17]. In fact to accomplish this comparison, we had to use almost 200 μg equivalents of the two proteins in the SDS-PAGE in order to make detectable provisions for the poor expression levels of the CaM-deficient iNOSfl protein. Fig 2, Panel A shows the comparative levels of iNOSfl dimer and monomer as manifested by such analysis using a monoclonal anti-iNOS antibody. The corresponding SDS-PAGE based CaM-immunoblots of equal amounts of the above purified iNOSfl proteins [in terms of iNOS protein content determined through anti-iNOS Western blot (Fig 2, Panel B)] using a monoclonal anti-CaM antibody, revealed the presence of CaM, as a probable evidence of its association with the iNOSfl protein co-expressed with CaM (Fig 2, Panel B). However, no CaM was detected in the iNOSfl protein expressed and purified in the absence of CaM (Fig 2, Panel B) confirming that the bacterial expression system used [*E*.*coli* BL21(DE3)] probably does not produce this protein. Such observations were indeed consistent with previous reports that CaM remains closely bound to the iNOSfl protein when they are expressed together [22,12].

Estimation of relative heme content of the two proteins both by a heme-specific fluorescent probe, PIA-514 (Fig 2C) as well as heme-CO adduct evaluation (Fig 2E and 2F and Table 1) of Western blot based equivalent amounts of iNOSfl proteins (Fig 2D), respectively revealed almost 12 to 10 folds higher heme presence in the CaM-replete iNOSfl compared to its CaM-deficient counterpart (Fig 2G). This observation contradicted previous reports that suggested that iNOSfl purified in the absence of CaM was essentially heme-free [12]. Moreover, the detectable presence of a dithionite reduced heme-CO adduct soret absorbance at 444 nm for the CaM-deficient iNOS protein (Fig 2F) revealed that the whole or part of its heme iron was also axially ligated to a cysteine thiol as is prominently observed in the normal and active CaM-replete iNOS (Fig 2E). Such results do establish that although the absence of CaM co-expression does compromise the level of heme-incorporation in the iNOSfl protein to a considerable extent, it does not render the enzyme completely heme-free nor does it completely restrain the enzyme from productively utilizing such low level of incorporated heme to form detectable levels of the NOS dimer.

**Table 1:** Relative Heme, Steady state NO synthesis and Arg and H_4_B K_m_ values for iNOSfl proteins expressed and purified in the presence and absence of CaM.

Protein	Heme (μM)	NO synthesis activity (min^-1^) per μg of protein	*K_m_ (Arg) at 25ºC(μM) per μg of protein	*K_m_ (H_4_B) at 25ºC(μM) per μg of protein
**iNOSfl (+ CaM)**	**50.45 ± 3.6**	**94 ± 3.2**	**3.7 ± 0.08**	**1.92 ± 0.55**
**iNOSfl (- CaM)**	**4.65 ± 0.38**	**10.8 ± 0.6**	**0.42 ± 0.06**	**0.21 ± 0.02**

*Apparent K_m_ values were derived from NO synthesis measurements taken at room temperature (25°C). All assays are described under *'Materials and Methods'*. Results shown are the mean and S.D. of three independent experiments done under similar conditions.

In order to check whether the modest amount of the NOS dimer in the CaM-deficient iNOSfl was active enough to sustain detectable NO synthesis, we subjected it along with the CaM-replete iNOSfl to the sensitive oxyhemoglobin assay (see *'Methods and Materials'*) using equivalent amounts of the purified proteins. Indeed, the iNOSfl protein expressed in the absence of CaM could manifest a NO production capability which was almost nine times less than its CaM-associated counterpart (Table 1). We further examined the relative K_m_ values for the substrate, L-Arg and the critical cofactor, H_4_B for the CaM-deficient and replete iNOS proteins through a NO-synthesis based evaluation of the same, and found that the K_m_ for L-Arg and H_4_B were respectively six and four times lower for the CaM-deficient enzyme compared to its CaM-bound form (Table 1). This indicated that the same factors that probably contributed to the poor level of heme incorporation in the newly synthesizing iNOSfl protein due to the absence of CaM, were probably involved in adversely affecting the binding of the substrate, L-Arg and the catalytically indispensible cofactor for iNOS, H_4_B to the CaM-deficient iNOSfl protein. Whether such effect is a consequence of poor heme incorporation or overall fallout of possible misfolding of the nascent iNOSfl protein subunits during its translational or post-translational assembly in the absence of CaM, needs to be further investigated. However, it is clear from the above observations that CaM has a partial role in imparting the required structure-function integrity to the iNOSfl oxygenase domain for executing accurate interactions with the major catalytic and functional stakeholders of iNOS for efficient NO synthesis.

**Fig 3:**
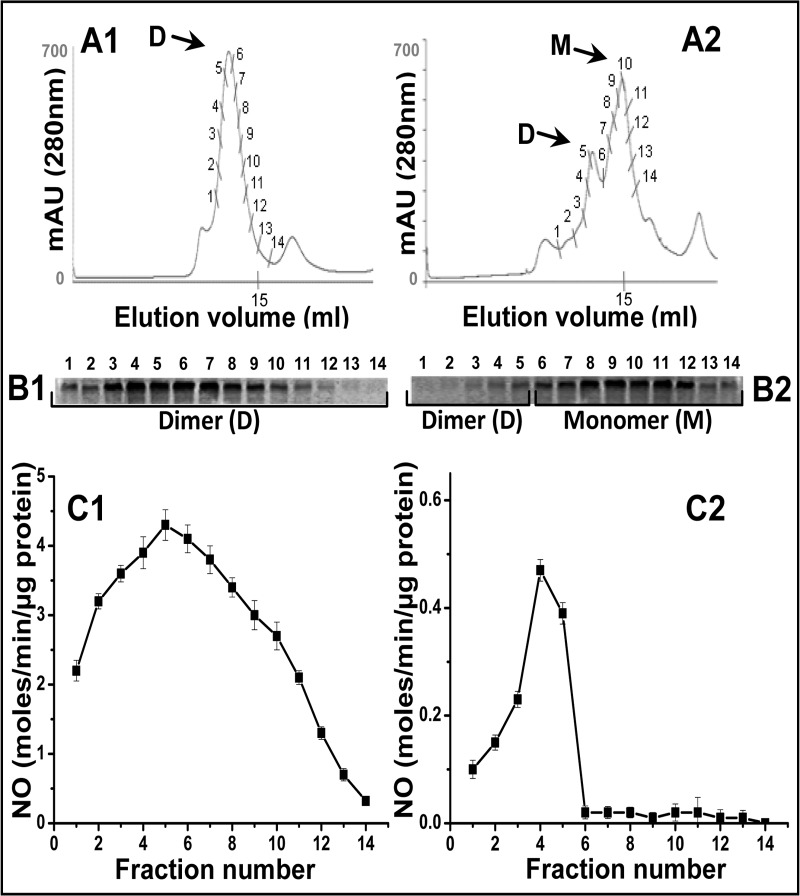
Evaluation of dimer-monomer content and corresponding catalytic activities of iNOSfl proteins co-expressed with and without CaM. Top panels depict the gel filtration chromatography profiles of the dimer and monomer of the purified iNOSfl proteins expressed in the presence (**Panel A1**) and absence (**Panel A2**) of CaM while the middle panel shows the corresponding SDS-PAGE based immunoblots of iNOS proteins depicting corresponding levels of the iNOS dimer and monomer in the indicated protein fractions (100 μl) for the iNOSfl proteins expressed with (**Panel B1**) and without (**Panel B2**) CaM. The bottom panel shows the NO synthesis activities of the same iNOSfl protein fractions measured through the Griess assay (*see 'Materials and Methods'*) against a sodium nitrite standard curve. Data shown are representative of three independent experiments done under similar conditions.

We further analyzed the two proteins through size exclusion chromatography or gel filtration to more precisely examine their relative dimer-monomer content as well as catalytic efficiency of their dimeric forms. Fig 3 shows the native gel-filtration profiles of the purified iNOSfl proteins expressed in the presence (Fig 3A1) and absence of CaM (Fig 3A2). As is apparent, the dimer content of the CaM-deficient iNOSfl protein (Fig 3A1) was significantly lower (28%) compared to its CaM-replete counterpart (Fig 3A2) which was almost completely dimeric. When the fractions collected through the above gel filtration analysis representing the dimeric and monomeric populations of the two proteins were subjected to SDS-PAGE (Fig 3B) as well as NO-synthesis assay (Fig 3C), discernibly poor dimer content (Fig 3B2) as well as comparative activities of such dimeric fractions (Fig 3C2) were observed for the CaM-deficient iNOSfl protein when compared to the CaM-rich iNOSfl (Fig 3B1 and 3C1). It is intriguing that the previous and possibly the first characterization study of such CaM-deficient iNOSfl protein did notice almost similar dimer content compared to us through the same analysis but unfortunately failed to register any activity for such dimeric fractions through their NOS activity assays [12]. One possibility for such discrepancy could be the use of apparently low amounts of the CaM-deficient iNOSfl protein in their study [12] for the NOS activity assays which compounded by the extremely poor activity of such protein did not perhaps allow them to register any detectable heme presence or NO-synthesis of such dimeric fractions or the purified protein, thus driving them to infer that the iNOSfl protein synthesized in the absence of CaM co-expression is ‘heme-free’ as well as ‘inactive’. The fact that we used almost 4–5 times more protein for such analysis compared to that used for heme-replete iNOS following standard assay protocols, was possibly a determining factor behind our overcoming any existing sensitivity barriers governing the detection thresholds of the techniques utilized for such assays and recording both the presence of heme and detectable NO synthesis in the CaM-free iNOSfl protein.

Although a functional heme-moiety (P450-heme) is essential for dimerization of NOS and rendering it catalytically active, the relation between the heme content, dimer level and the NO formation activity is not found to be stoichiometric for the NOS enzyme largely because there are several other factors involved in dimerization as well as catalysis of NOS besides heme (i.e L-Arg, H_4_B etc.) [8]. In fact, it is even more challenging to interpret such relation in the case of the CaM-deficient iNOS which despite having a normal reductase domain has a functionally defective oxygenase domain not only in terms of its poor heme content but its affinity for the substrate, L-Arg as well as the essential cofactor H_4_B when compared to the CaM-replete iNOSfl protein, which shows a more definitive pattern of correlation between its heme content, dimerization capability and catalytic activity.

**Fig 4:**
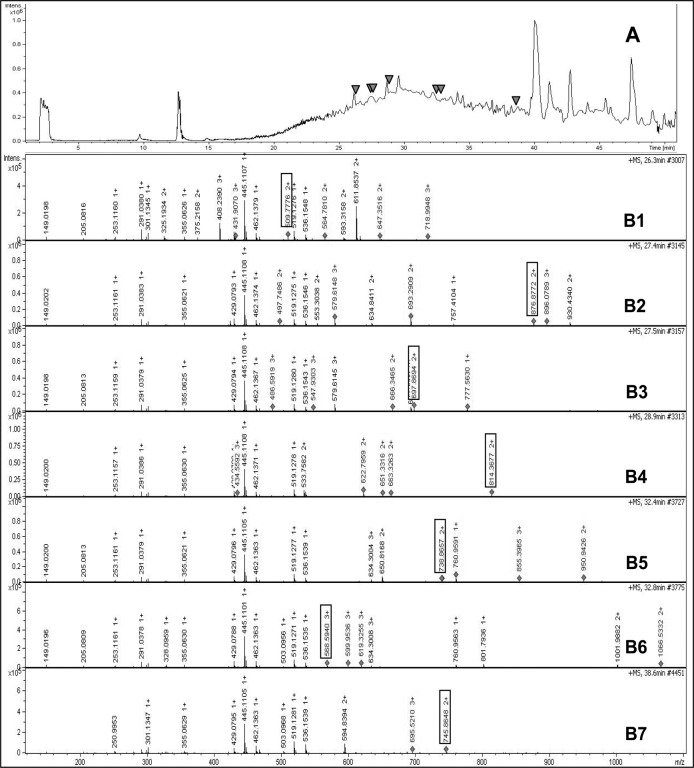
Nano LC-MS/MS analysis of iNOSfl protein expressed and purified in the absence of CaM. In-solution tryptic digest of iNOS expressed in absence of CaM was separated on a C18 reversed-phase nano column at a flow rate of 5000 nl/min. The effluent was fed directly on a maXis impact mass spectrometer (Bruker Daltonics). The Flex Analysis version 3.4 was used to calculate values for all expected tryptic peptides. Mass chromatograms spanning the m/z (mass-to-charge ratio) ranging from 50 to 2200 were generated for each expected mass. **Panel A** shows the base peak chromatogram obtained by plotting the intensity of the most intense ion in a spectrum versus the elution time. 8146 scans were collected. Arrowheads in **Panel A** indicate the peaks whose detailed mass spectra are depicted in **Panels B1 to B7**. The ions corresponding to charge states of seven different peptides (**Panels B1—B7**) (509.7, 568.5, 697.8, 738.8, 745.8, 814.3, 876.8) could be uniquely assigned to predicted products of the tryptic digest of iNOS. Data are representative of two experiments done under independent conditions.

**Fig 5:**
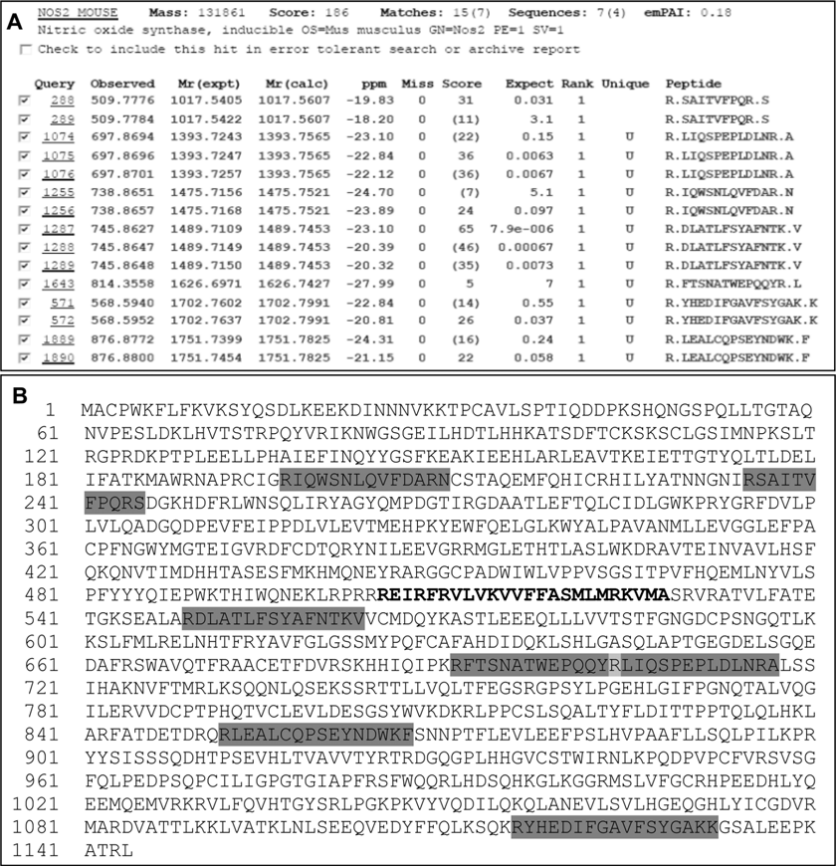
Nano LC-MS/MS peptide report of iNOSfl protein expressed and purified in the absence of CaM and corresponding peptide match with established protein sequence of CaM-replete iNOSfl protein. **Panel A** shows the LC-MS/MS peptide report obtained after in-solution digestion for the iNOSfl protein expressed and purified in absence of CaM corresponding to the detected peptides delineated in Fig 4, **Panels B1 to B7** where 509.7 corresponds to (R)SAITVFPQR(S) (residues 235–245), 568.5 to (R)YHEDIFGAVFSYGAK(K) (residues 1116–1132), 697.8 to (R)LIQSPEPLDLNR(A) (residues 704–717), 738.8 to (R)IQWSNLQVFDAR(N) (residues 197–210), 745.8 to (R)DLATLFSYAFNTK(V) (residues 549–563), 814.3 to (R)FTSNATWEPQQYR(L) (residues 691–705) and 876.8 to (R)LEALCQPSEYNDWK(F) (residues 852–867). **Panel B** represents the amino acid sequence of inducible nitric oxide synthase (Uniprot) and the highlighted regions (dark grey) depict the seven matched peptides **(Panel A)** after LC/MS analysis along with the CaM binding domain (bold). Data are representative of two experiments done under independent conditions.

To figure out whether the iNOSfl protein purified in the absence of CaM is structurally intact or has the complete functional sequence compared to that of the CaM-replete iNOSfl protein, we subjected the purified protein to LC-MS/MS based peptide mapping analysis. Fig 4 shows that the in-solution tryptic digest of the purified CaM-deficient iNOSfl protein when subjected to such analysis generated mass chromatograms for at least seven peptides that could be attributed to the murine iNOSfl protein (Fig 4 and Fig 5A) which virtually encompassed almost the entire sequence of the established sequence of the full-length version of the iNOSfl protein (Fig 5B). This indicated that the CaM-free iNOSfl protein indeed had the same complete sequence of the functional CaM-replete iNOSfl protein [13]. Moreover, no peptide match with the CaM protein was found through the same analysis, further confirming the absence of CaM association with the iNOSfl protein expressed and purified in the absence of CaM expressing plasmids in *E*.*coli*.

**Fig 6:**
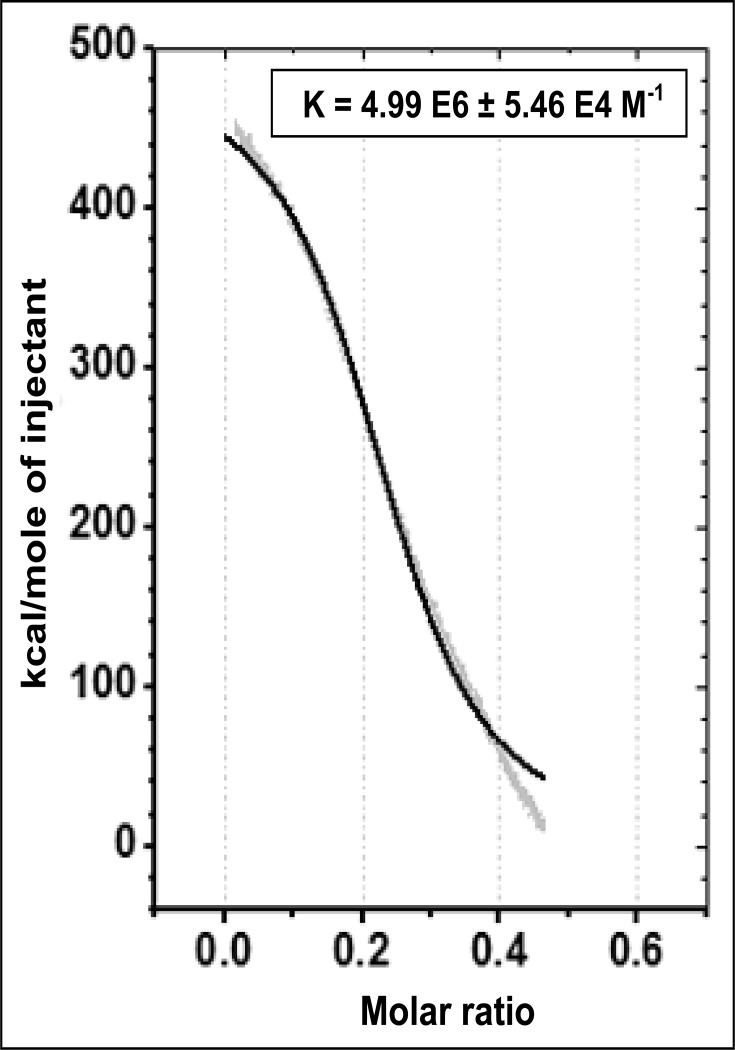
Single Injection Mode Isothermal Calorimetric evaluation of CaM binding to the CaM-free iNOSfl protein. Plot shows the enthalpy (kcal/mol of injectants) change obtained through single injection based mixing of 20 μM CaM-free iNOSfl with 8 μM of CaM. Data analysis through one site curve fitting is reported for the binding of CaM to iNOSfl expressed and purified in absence of CaM. Results shown are representative of three independent experiments done under similar conditions.

To test whether this CaM-deficient iNOSfl protein was indeed capable of binding CaM, we further subjected it to Isothermal Titration Calorimetric CaM binding assay. Fig 6 clearly shows that the CaM could bind to this protein with observable high affinity (K = 4.99E6±5.46E4 M^-1^) and Δ H = 4.887E5 ±1109 ±cal/mol, Δ S = 1.67E3 cal/mol/deg and Δ G = - 9.21E3 cal/mol establishing the possible presence of a free CaM-binding site in such intact iNOSfl protein purified without CaM co-expression as well as fairly strong binding capacity of such motif to CaM. More importantly, such revelation also creates necessary avenues for studying the actual affinity of CaM for the iNOSfl protein which is generally expressed in the presence of CaM in its stable and active CaM-bound form.

While analyzing the functional characteristics of the reductase domain of the CaM-deficient iNOSfl enzyme, we also found that the total flavin content, cytochrome c activity and ferricyanide activity of the CaM-free iNOSfl protein was almost similar to that observed for the CaM-bound iNOSfl (Table 2) much in contrast to that reported by Wu *et*.*al* [12]. This indicates that co-expression with CaM is perhaps not as essential for maintaining the structural and functional integrity of the reductase domain of iNOS during its translational assembly or post-translational folding events as much as the oxygenase domain, which is profoundly affected by the absence of CaM both in terms of its ability to retain and utilize functionally essential cofactors like heme and H_4_B as well as the substrate, L-Arg during the synthesis of the iNOSfl protein. This perhaps also exempts the possible role of the reductase domain in the poor functional rendering of the CaM-deficient iNOSfl protein and helps to particularly implicate the oxygenase domain as the primary ‘culprit’ for the observed functional shortcomings of the enzyme when it is produced without the support of CaM.

**Table 2:** Comparative Flavin content, Cytochrome c reduction and Ferricyanide reduction rates of iNOSfl proteins expressed and purified in the presence and absence of CaM.

Protein	Flavin content (mol/mol of iNOSfl protein)	Cytochrome c reductionrate (min^-1^)	Ferricyanide reductionrate (min^-1^)
**iNOSfl (+ CaM)**	**1.67 ± 0.11**	**9832 ± 92**	**9985 ± 115**
**iNOSfl (- CaM)**	**1.58 ± 0.15**	**8560 ± 105**	**9125 ± 98**

Measurements were made at 25°C (room temperature). Values represent the mean S.D. of three independent measurements done under identical conditions.

Our study does concur with the observation that CaM is important for translational assembly and perhaps efficient folding of the iNOS protein for rendering it catalytically active and that without CaM the iNOS enzyme does become functionally handicapped in several respects [2,9,12]. However, our present characterization of the CaM-free iNOS tends to challenge the claim that deprivation of CaM during the synthesis of iNOS leads to the generation of a catalytically ‘dead’ protein [12]. Indeed the iNOSfl protein expressed in the absence of CaM in our study, albeit with all its shortcomings, showed humble NO synthesis activity after binding with CaM *in vitro*, a property which can be effectively utilized to understand the precise role of post-translational CaM binding to the iNOS protein and more importantly to answer the still unaddressed question as to why the constitutive NOS isoforms, nNOS and eNOS do not similarly require CaM for their translational assembly and are only dependent on it perhaps for mobilizing inter-domain electron transfer [3,11].

In all, our study revisits characterization of the CaM-deficient iNOSfl in an attempt to fill up the observational gaps in a previous study of the same protein and suggests the future utilization of the protein as a tool to better understand the role of CaM in the functional assembly of the iNOS enzyme as well as the factors governing differential response and requirement of CaM binding to the three different NOS isoforms for their synthetic and catalytic needs.
