# Correction: Characterization of Calmodulin-Free Murine Inducible Nitric-Oxide Synthase

**DOI:** 10.1371/journal.pone.0130222

**Published:** 2015-06-08

**Authors:** The PLOS ONE Staff

The corresponding author’s email is listed incorrectly. The correct email address is pandak66@yahoo.co.uk.
