# Correction: Characterization of Calmodulin-Free Murine Inducible Nitric-Oxide Synthase

**DOI:** 10.1371/journal.pone.0240744

**Published:** 2020-10-09

**Authors:** Latika Nagpal, Koustubh Panda

The authors issue this Correction to address the following issues that were noted after this article [1] was published:

- In Fig 1A, there appear to be vertical discontinuities between lanes, the lanes were mislabeled, and the image was stretched vertically from its original size. The authors commented that the apparent discontinuities resulted from a streaking effect observed between lanes on the original gel image. The original image supporting the published figure panel was not provided, but an updated Fig 1 is provided here, in which panel A is replaced with replicate data. The raw image supporting the updated version of Fig 1A is in S1 File, and additional replication data supporting this experiment are in S2 File.
- Questions were raised about the lack of loading control data reported in [1] for the blots shown in Figs 2A, 2B, and 2D. Figs 2A-2D show results of western blot (A, B, D) and SDS-PAGE (C) experiments in which inputs were total bacterial extract (A) or affinity column-purified iNOS (B-D). The authors clarified that for western blot experiments, membranes were stained with Ponceau S after protein electro-transfer and prior to antibody probing both to confirm the efficacy of protein transfer as well as to be evaluated as loading controls. Ponceau S staining results and raw blot/gel image data for the reported experiments are provided in S3 File.

**Fig 1:**
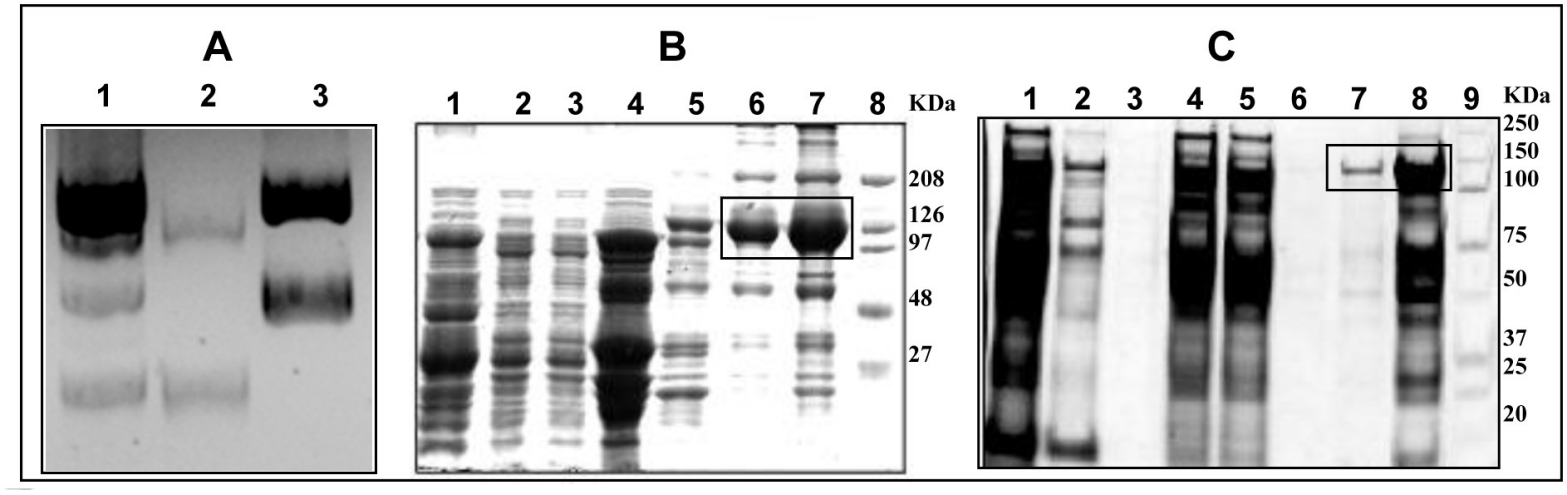
DNA and protein purification profiles of bacterially expressed recombinant iNOSfl in the presence and absence of CaM. **Panel A** shows the plasmid DNA profiles in BL21(DE3) E. coli cells containing iNOSfl expressing plasmids along with (lane 1) or without (lane 3) CaM expressing plasmids against only the CaM plasmid DNA (lane 2). 0.5 μg each of the isolated DNA samples were loaded in 0.8% agarose gel in which the DNA size ranged between 9.5 to 3.5 kb. **Panels B and C** show the 10% SDS-PAGE profiles of the protein fractions collected at each step of purification of iNOSfl co-expressed with CaM (B) and without CaM (C) respectively. SDS-PAGE protein profiles represent 10 μl of the indicated fractions after staining with Coomassie blue. **Panel B** depicts the protein profiles of fractions collected at different stages of purification of iNOSfl in the presence of CaM comprising of the suspension of the ammonium sulphate cut pellet of the bacterial lysate (Lane 1); Ni-NTA column flow-through (Lane 2); Ni-NTA imidazole eluate (Lane 3); ADP column flow-through (Lane 4); ADP column zero wash (Lane 5); ADP column NADPH eluate (Lane 6) and concentrated NADPH eluate of the purified iNOSfl protein expressed with CaM (Lane 7) along with standard molecular weight markers (Lane 8). Similarly, **Panel C** shows the protein profiles of fractions collected at different stages of purification of iNOSfl in the absence of CaM, namely the suspension of the ammonium sulphate cut pellet of the bacterial lysate (Lane 1); Ni-NTA column flow-through (Lane 2); Ni-NTA column zero wash (Lane 3); Imidazole eluate (Lane 4); ADP column flow-through (Lane 5); ADP column zero wash (Lane 6); ADP column NADPH eluate (Lane 7) and concentrated NADPH eluate of the purified iNOSfl (-CaM) protein (Lane 8) along with standard molecular weight markers (Lane 9). The pure iNOSfl bands are boxed in Panels B and C.

As demonstrated in Fig 2A and S5 File, the level of iNOSfl was observed to be substantially lower when the protein was expressed in the absence of CaM than in its presence. Higher levels of degraded iNOSfl proteins were observed in the iNOSfl preparations expressed without CaM. Equal amounts of lysate were loaded in lanes 1 & 2 for this experiment.

For Figs 2B-2D, different amounts of purified iNOSfl proteins were loaded in the plus versus minus CaM lanes so as to obtain approximately equivalent iNOSfl levels (130 KD bands) across lanes within each gel or blot. Loading volumes needed to achieve equivalent iNOSfl levels and were determined through standardization experiments for which data were not reported in the article. The iNOS blots in Fig 2B and 2D served as the controls for the CaM blot in Fig 2B and the heme gel in Fig 2C, respectively. For each of these experiments, duplicate gels were prepared for the control and experimental assays by loading the same purified iNOSfl proteins on parallel gels.

- In Fig 3, the iNOSfl western blot in panel B1 errantly reports a horizontally flipped version of the western blot in panel B2. The authors note that this was due to a figure assembly error. This is corrected in the updated version of Fig 3 provided here. The original blots underlying these panels are provided in S4 and S5 Files.
- The article’s Data Availability statement says, “All relevant data are within the paper.” The underlying data were not included with [1] but are provided herein S1–S9 Files. All original data are available except those supporting Fig 2D.

**Fig 3:**
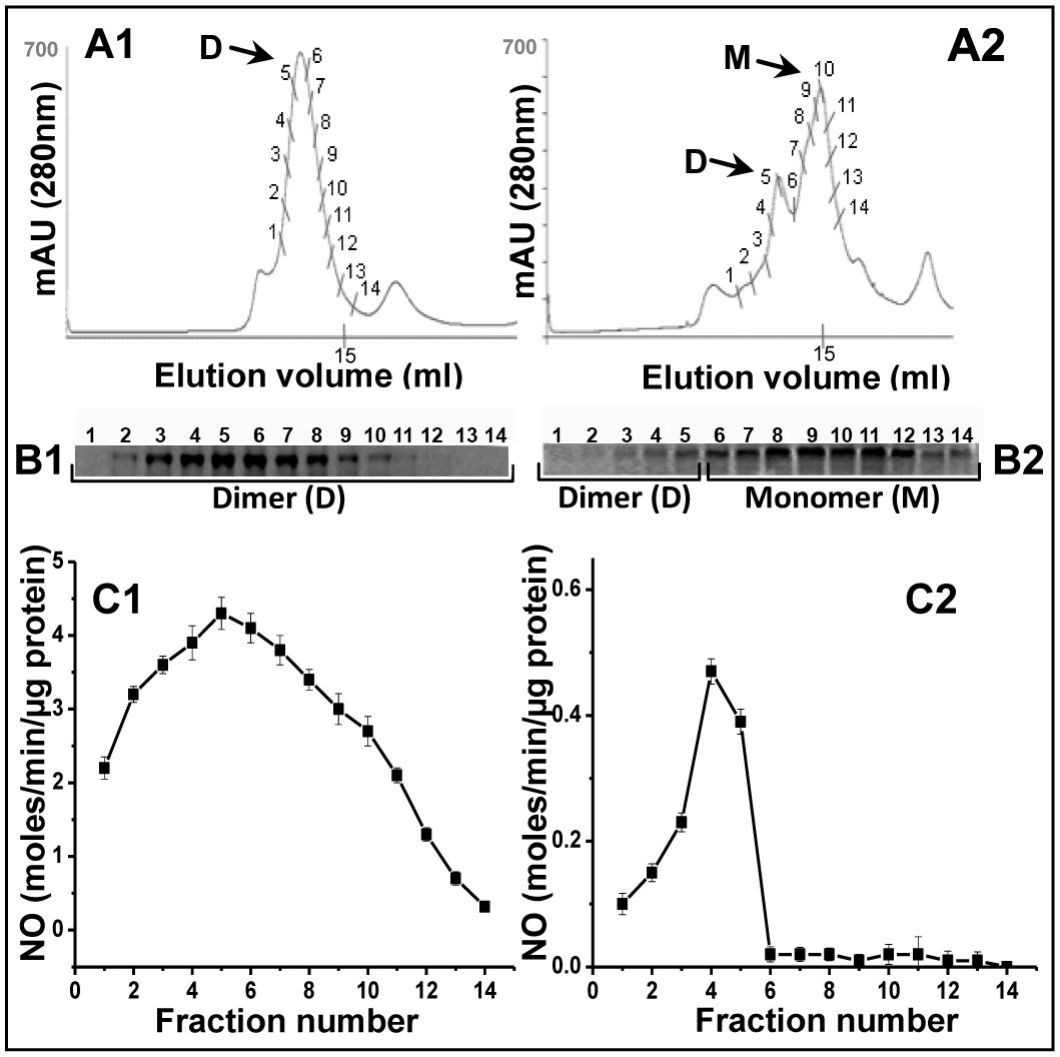
Evaluation of dimer-monomer content and corresponding catalytic activities of iNOSfl proteins co-expressed with and without CaM. Top panels depict the gel filtration chromatography profiles of the dimer and monomer of the purified iNOSfl proteins expressed in the presence (**Panel A1**) and absence (**Panel A2**) of CaM while the middle panel shows the corresponding SDS-PAGE based immunoblots of iNOSfl proteins depicting corresponding levels of the iNOSfl dimer and monomer in the indicated protein fractions (100 μl) for the iNOSfl proteins expressed with (**Panel B1**) and without (**Panel B2**) CaM. The bottom panel shows the NO synthesis activities of the same iNOSfl protein fractions measured through the Griess assay (see ‘Materials and Methods’ in [1]) against a sodium nitrite standard curve. Data shown are representative of three independent experiments done under similar conditions.

A member of *PLOS ONE*’s Editorial Board reviewed the updated figures, supporting data files, and information provided above regarding western blot controls and advised that they address the previous concerns and errors in the original manuscript. The Academic Editor commented that the corrections do not change the article’s conclusions.

The authors apologize for the errors in the published article.

## Supporting information

S1 File Original image supporting the replacement results shown in the revised version of Fig 1A. (PPT)

S2 File Replicate data supporting Fig 1A. (TIF)

S3 File Underlying data supporting Fig 2. The blot image provided for Fig 2D is a digital image of the original blot reported in the published figure; levels were adjusted in the image file so that band intensities would align approximately with bands observed by the Ponceau S staining. The original film from the Fig 2D blot experiment is no longer available.
(ZIP)

S4 File Original image data supporting Fig 3B1. (TIF)

S5 File Original image data supporting Fig 3B2. (JPG)

S6 File Underlying data supporting Fig 3A1, 3A2, 3C1 and 3C2. (ZIP)

S7 File Raw data supporting Fig 6. (PDF)

S8 File Raw data supporting Table 1. (ZIP)

S9 File Raw data supporting Table 2. (ZIP)
