# Supplementary figures and images for: Correction: Characterization of Calmodulin-Free Murine Inducible Nitric-Oxide Synthase
Source: PLoS One. 2020 Oct 9;15(10):e0240744. doi: 10.1371/journal.pone.0240744 (PMC7546496; doi:10.1371/journal.pone.0240744)

## Slide 1
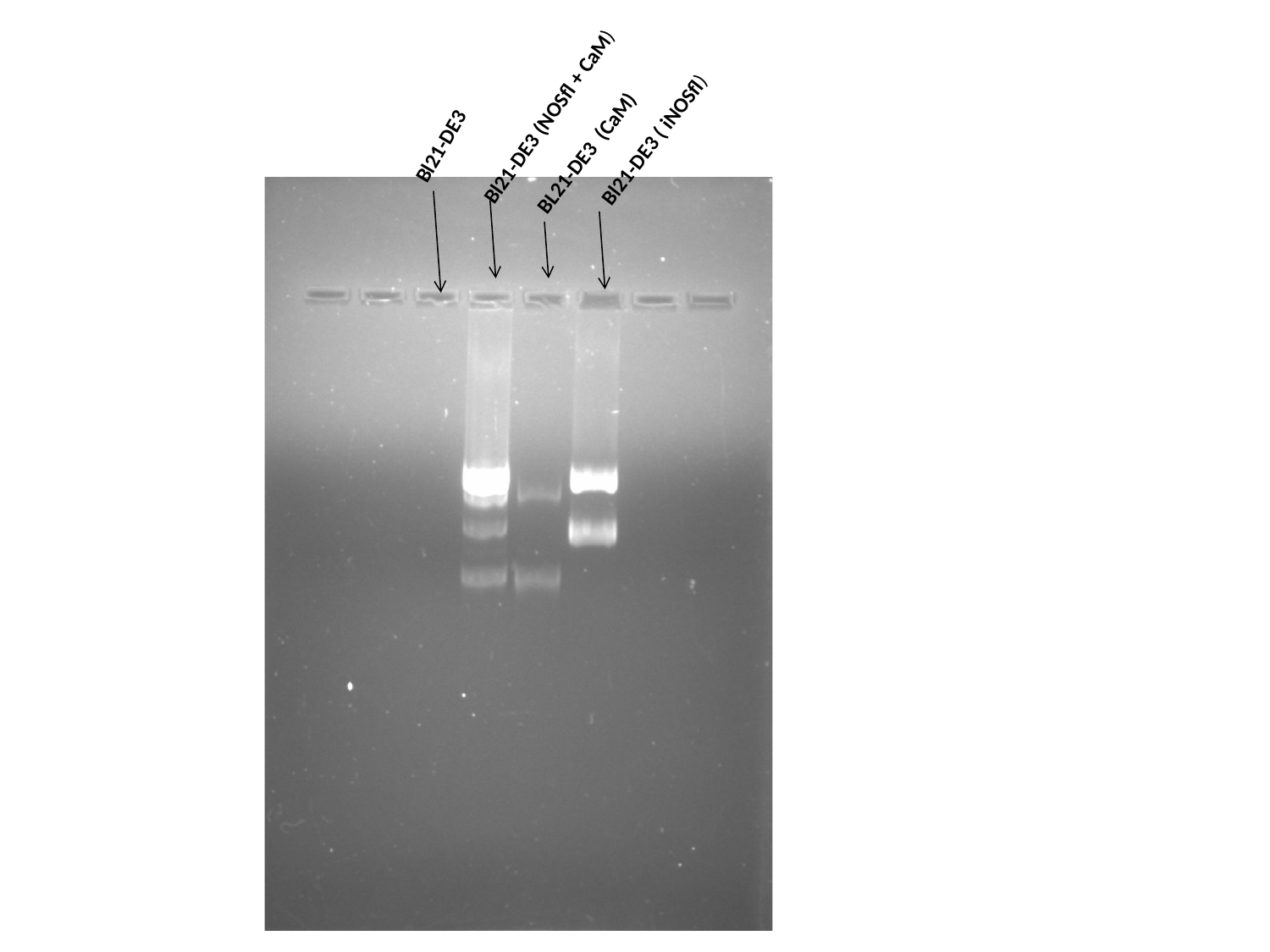

Bl21-DE3
Bl21-DE3 (NOSfl + CaM)
Bl21-DE3 ( iNOSfl)
BL21-DE3 (CaM)

Supplement: S1 File — (PPT) [file pone.0240744.s001.ppt]

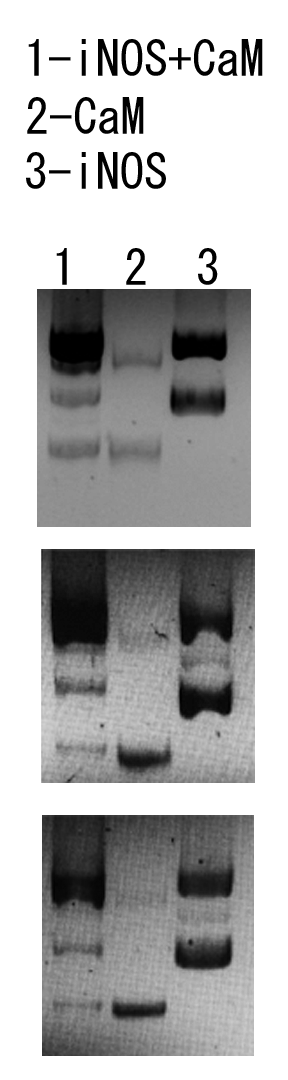

Supplement: S2 File — (TIF) [file pone.0240744.s002.tif]

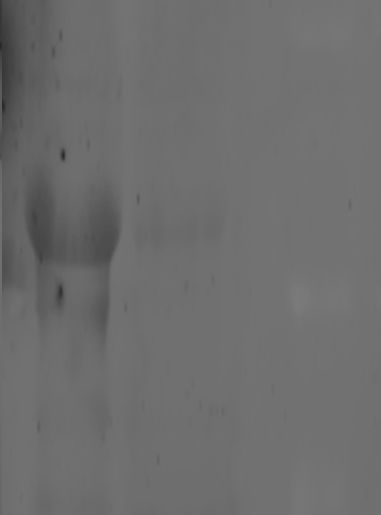

Supplement: S3 File — The blot image provided for Fig 2D is a digital image of the original blot reported in the published figure; levels were adjusted in the image file so that band intensities would align approximately with bands observed by the Ponceau S staining. The original film from the Fig 2D blot experiment is no longer available. (ZIP) [file pone.0240744.s003.zip › S5 File/Attachment#5_FIG2C_heme_stain_PIA_RAW_DATA.tif]

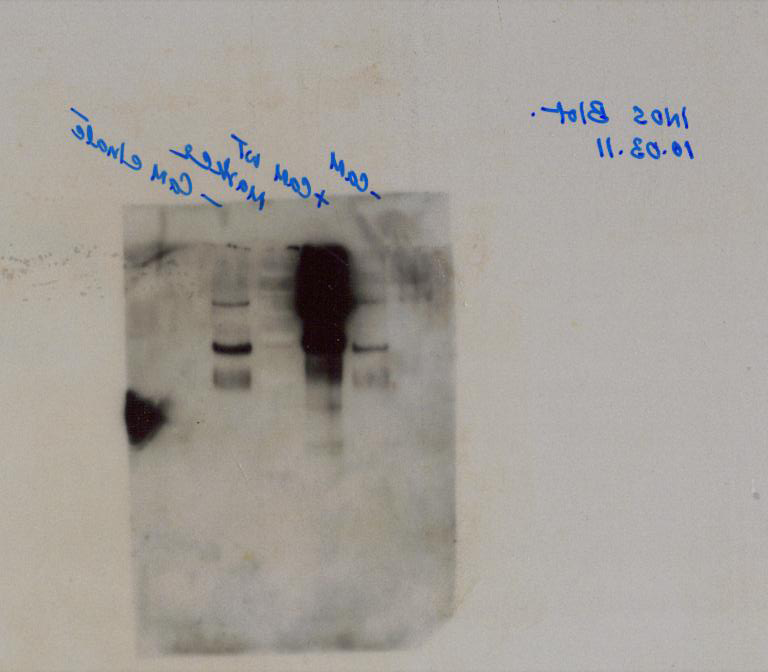

Supplement: S3 File — The blot image provided for Fig 2D is a digital image of the original blot reported in the published figure; levels were adjusted in the image file so that band intensities would align approximately with bands observed by the Ponceau S staining. The original film from the Fig 2D blot experiment is no longer available. (ZIP) [file pone.0240744.s003.zip › S5 File/Attachment#6_FIG2A_iNOS_Dimer_Monomer_RAW_DATA.tif]

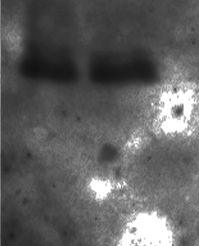

Supplement: S3 File — The blot image provided for Fig 2D is a digital image of the original blot reported in the published figure; levels were adjusted in the image file so that band intensities would align approximately with bands observed by the Ponceau S staining. The original film from the Fig 2D blot experiment is no longer available. (ZIP) [file pone.0240744.s003.zip › S5 File/Attachment#8_FIG2B_iNOS_RAW_DATA.tif]

1 2 1 2 1 2

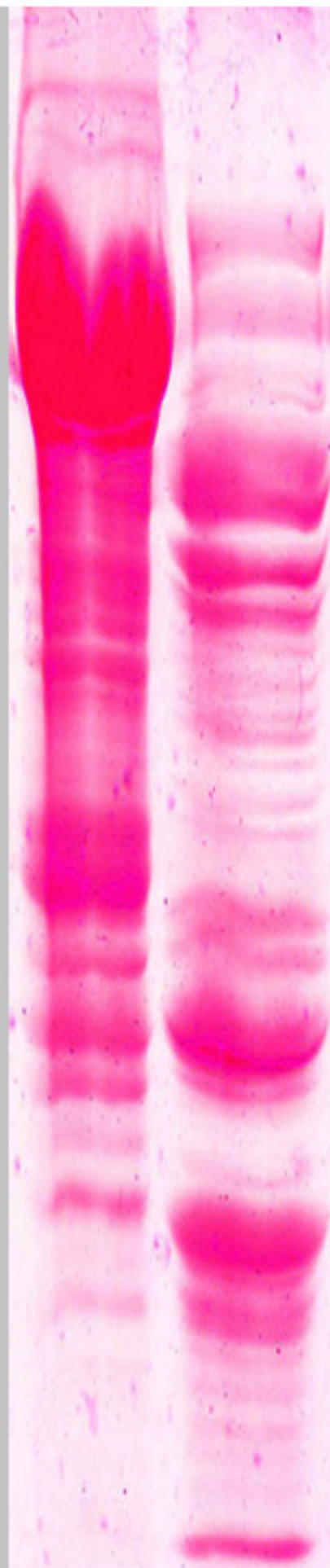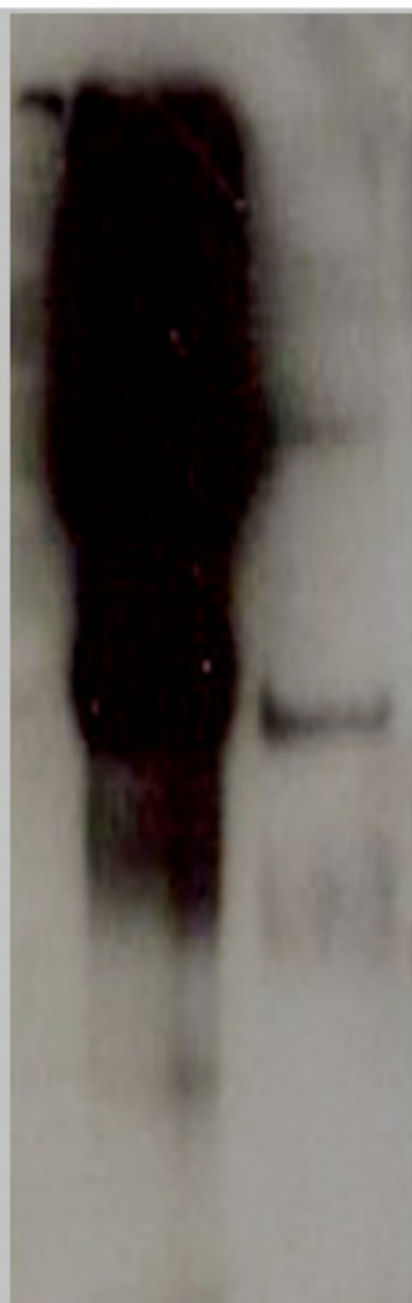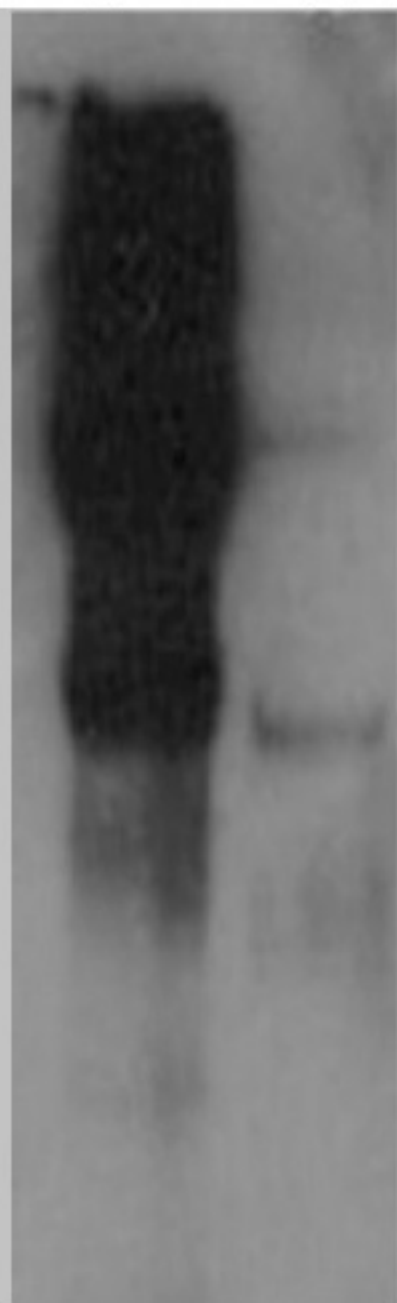

iNOS BLOT

FIG 2A

PONCEAU

Supplement: S3 File — The blot image provided for Fig 2D is a digital image of the original blot reported in the published figure; levels were adjusted in the image file so that band intensities would align approximately with bands observed by the Ponceau S staining. The original film from the Fig 2D blot experiment is no longer available. (ZIP) [file pone.0240744.s003.zip › S5 File/Fig.2A_Plos_One_iNOS_Lysates_Ponceau_Stain.pdf]

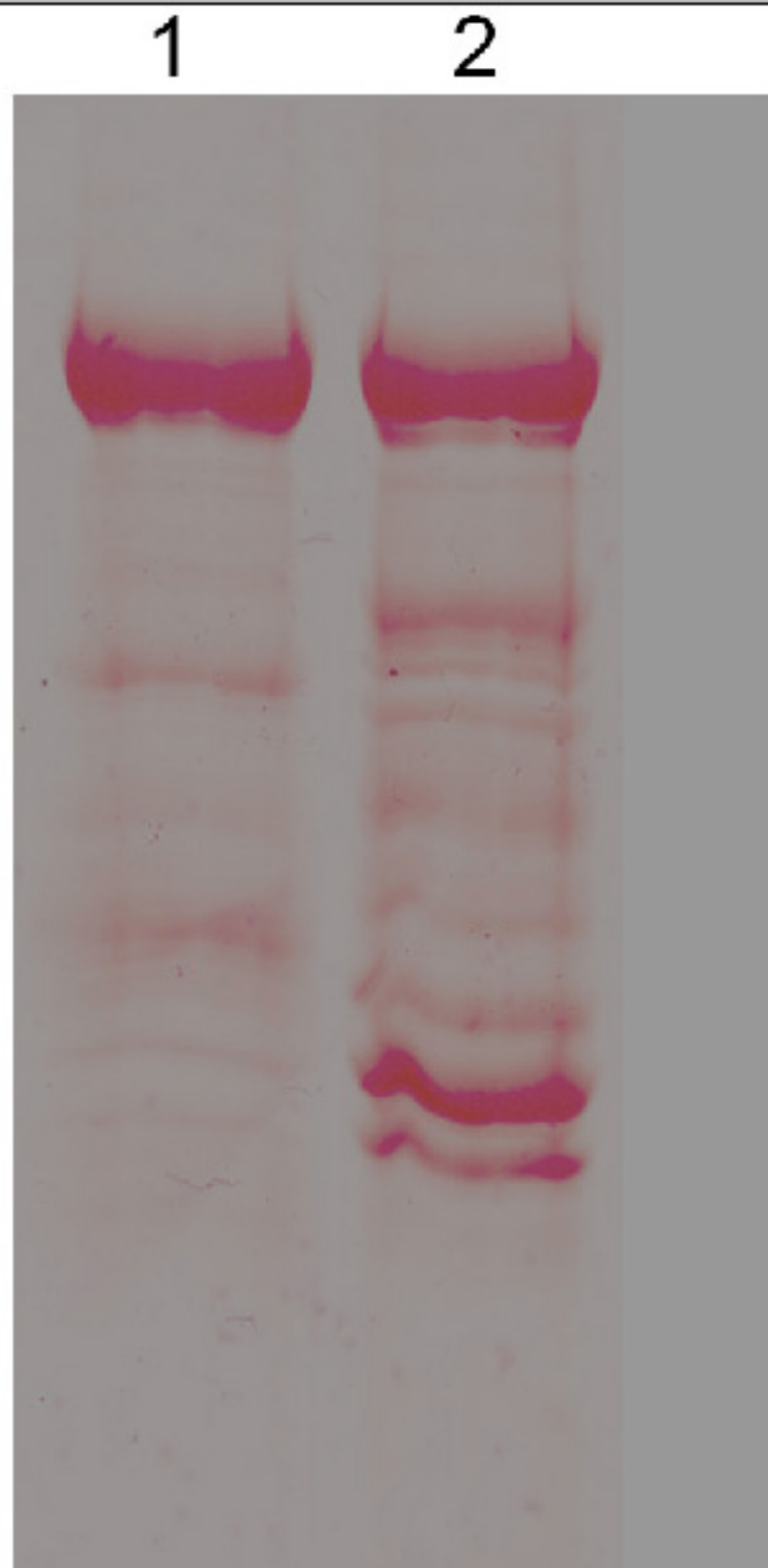

PONCEAU

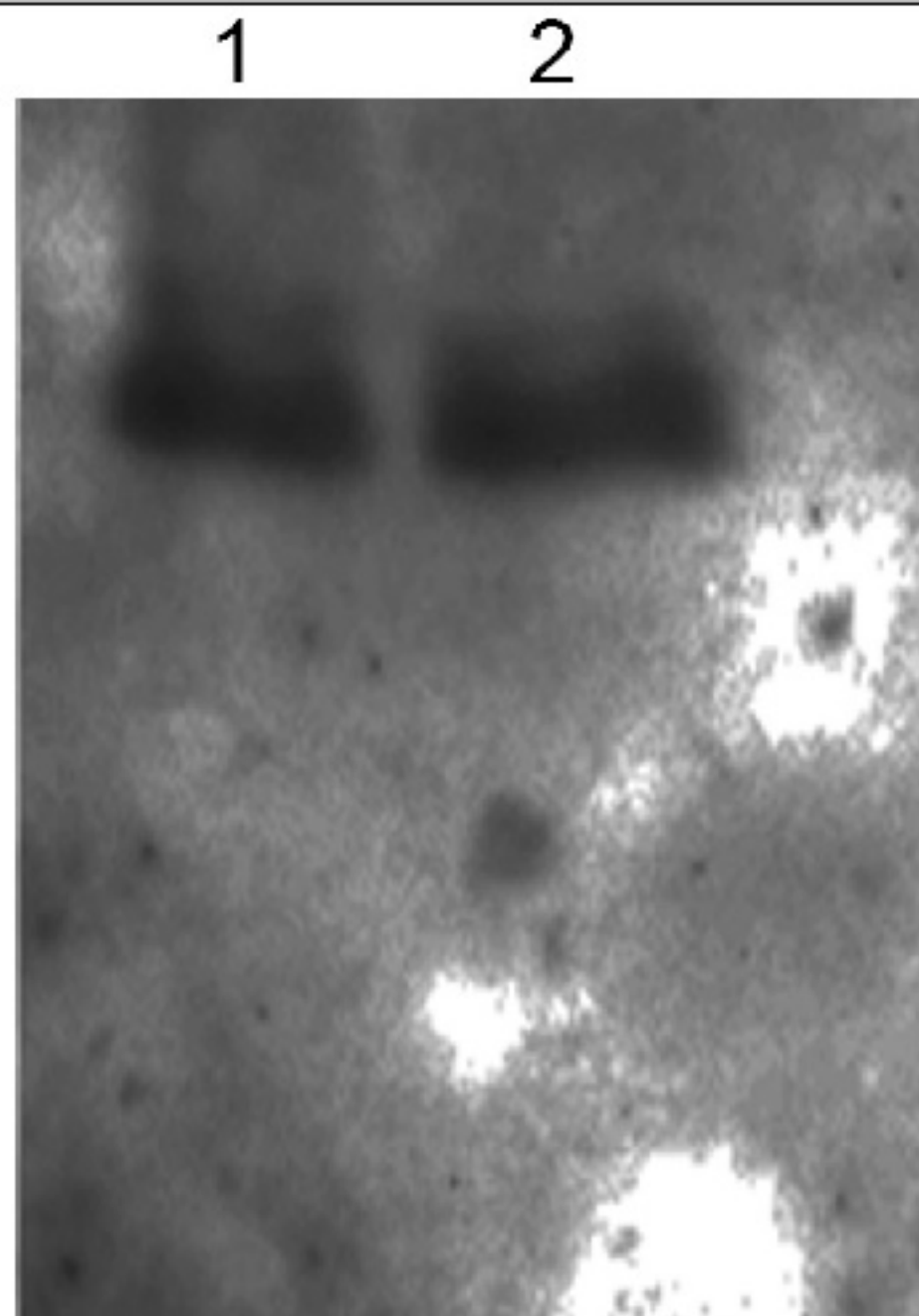

iNOS BLOT

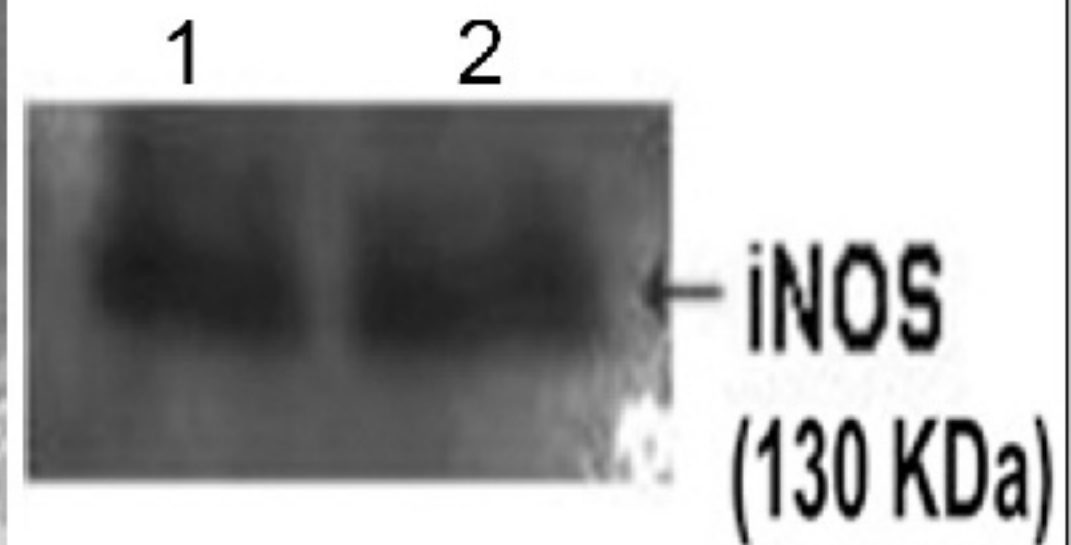

Fig 2B

Supplement: S3 File — The blot image provided for Fig 2D is a digital image of the original blot reported in the published figure; levels were adjusted in the image file so that band intensities would align approximately with bands observed by the Ponceau S staining. The original film from the Fig 2D blot experiment is no longer available. (ZIP) [file pone.0240744.s003.zip › S5 File/Fig.2B_Plos_One_iNOS_Ponceau_Stain.pdf]

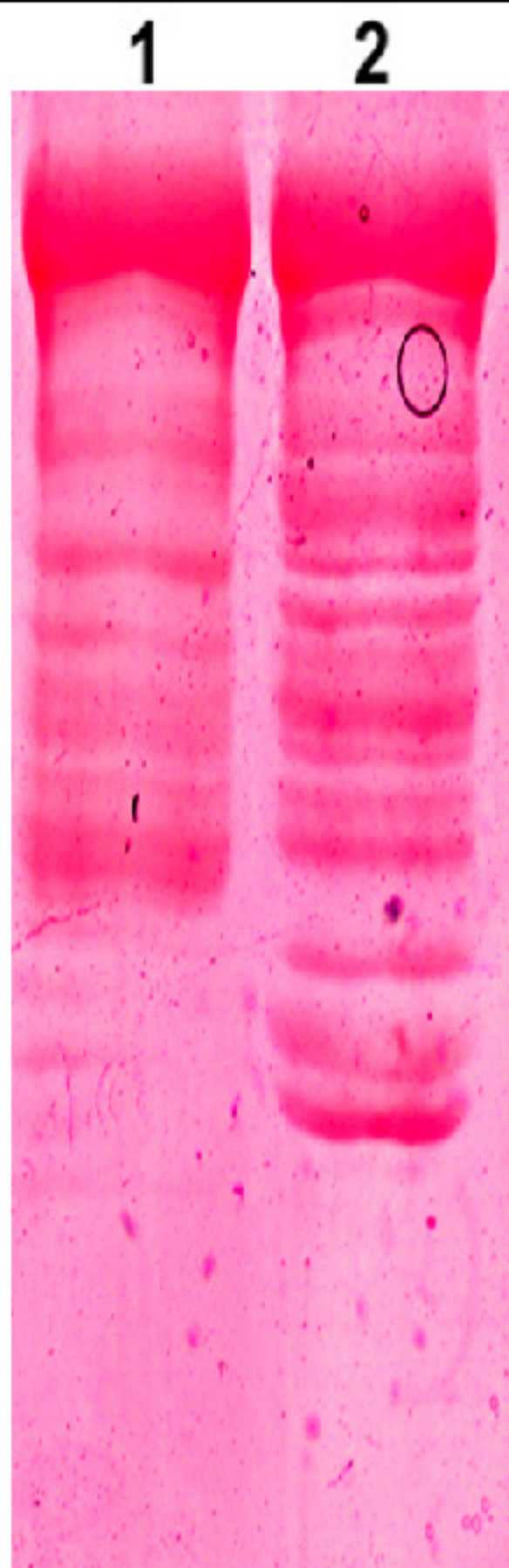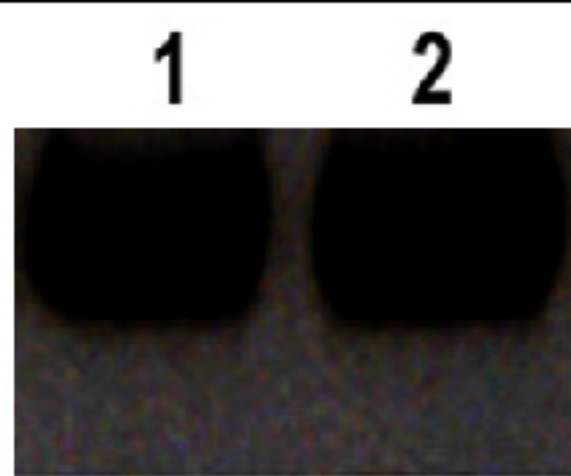

iNOS-BLOT  
(MIMIC)

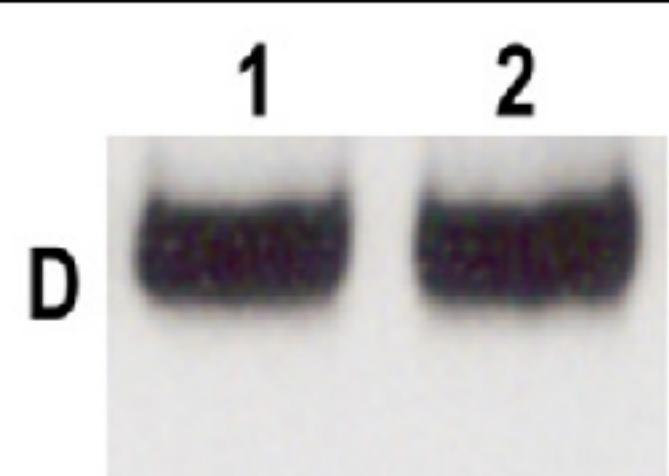

FIG. 2D

Supplement: S3 File — The blot image provided for Fig 2D is a digital image of the original blot reported in the published figure; levels were adjusted in the image file so that band intensities would align approximately with bands observed by the Ponceau S staining. The original film from the Fig 2D blot experiment is no longer available. (ZIP) [file pone.0240744.s003.zip › S5 File/Fig.2D_Plos_One_iNOS_Ponceau_Stain.pdf]

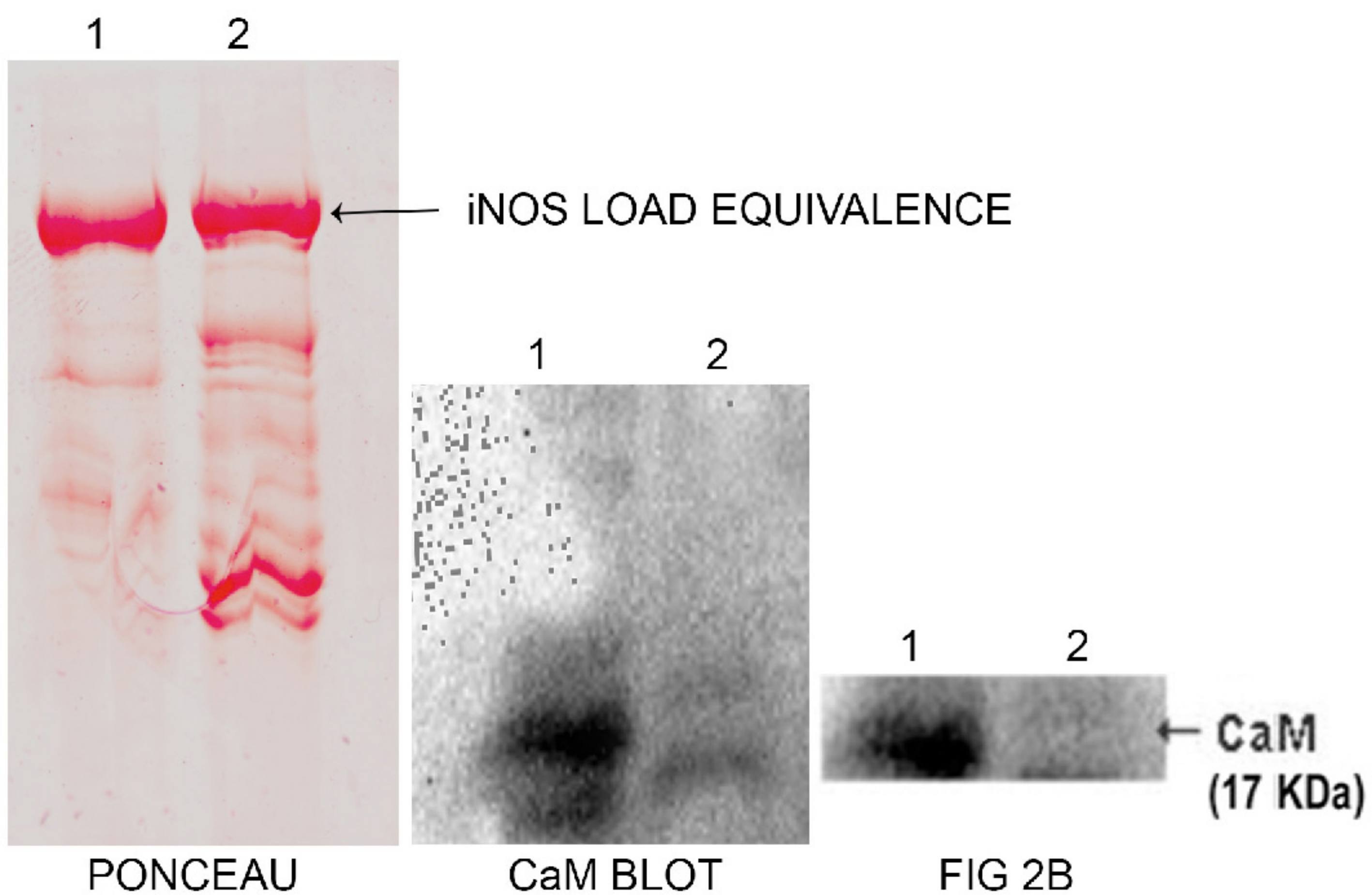

Supplement: S3 File — The blot image provided for Fig 2D is a digital image of the original blot reported in the published figure; levels were adjusted in the image file so that band intensities would align approximately with bands observed by the Ponceau S staining. The original film from the Fig 2D blot experiment is no longer available. (ZIP) [file pone.0240744.s003.zip › S5 File/Fig_2B_Plos_One_CaM_Ponceau_Stain.pdf]

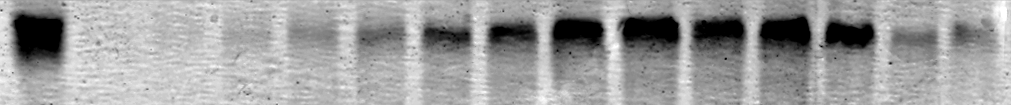

Supplement: S4 File — (TIF) [file pone.0240744.s004.tif]

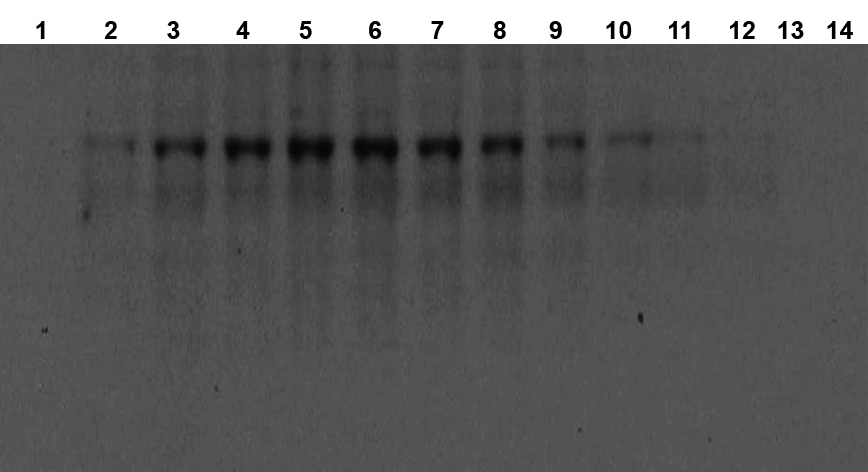

Supplement: S5 File — (JPG) [file pone.0240744.s005.jpg]

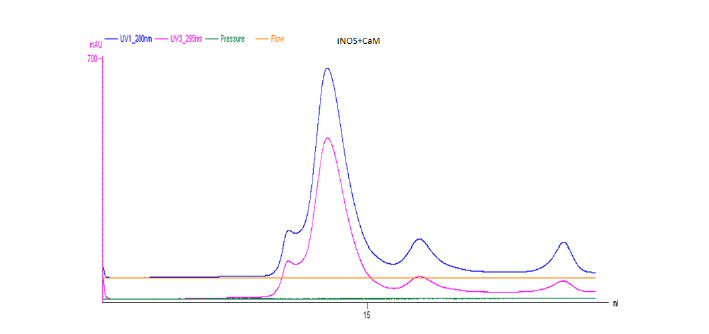

Supplement: S6 File — (ZIP) [file pone.0240744.s006.zip › S6 File/Attachment#12_Original_FPLC_Profile_iNOS+CaM.tif]

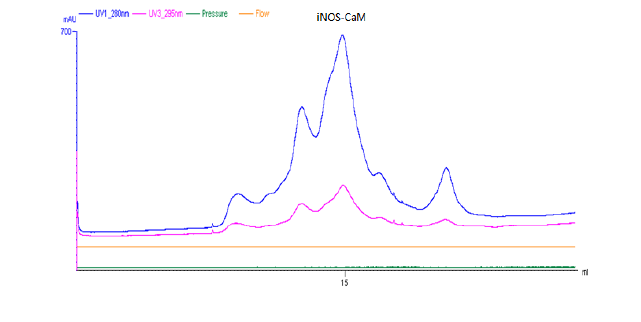

Supplement: S6 File — (ZIP) [file pone.0240744.s006.zip › S6 File/Attachment#13_Original_FPLC_Profile_iNOS_without_CaM.tif]

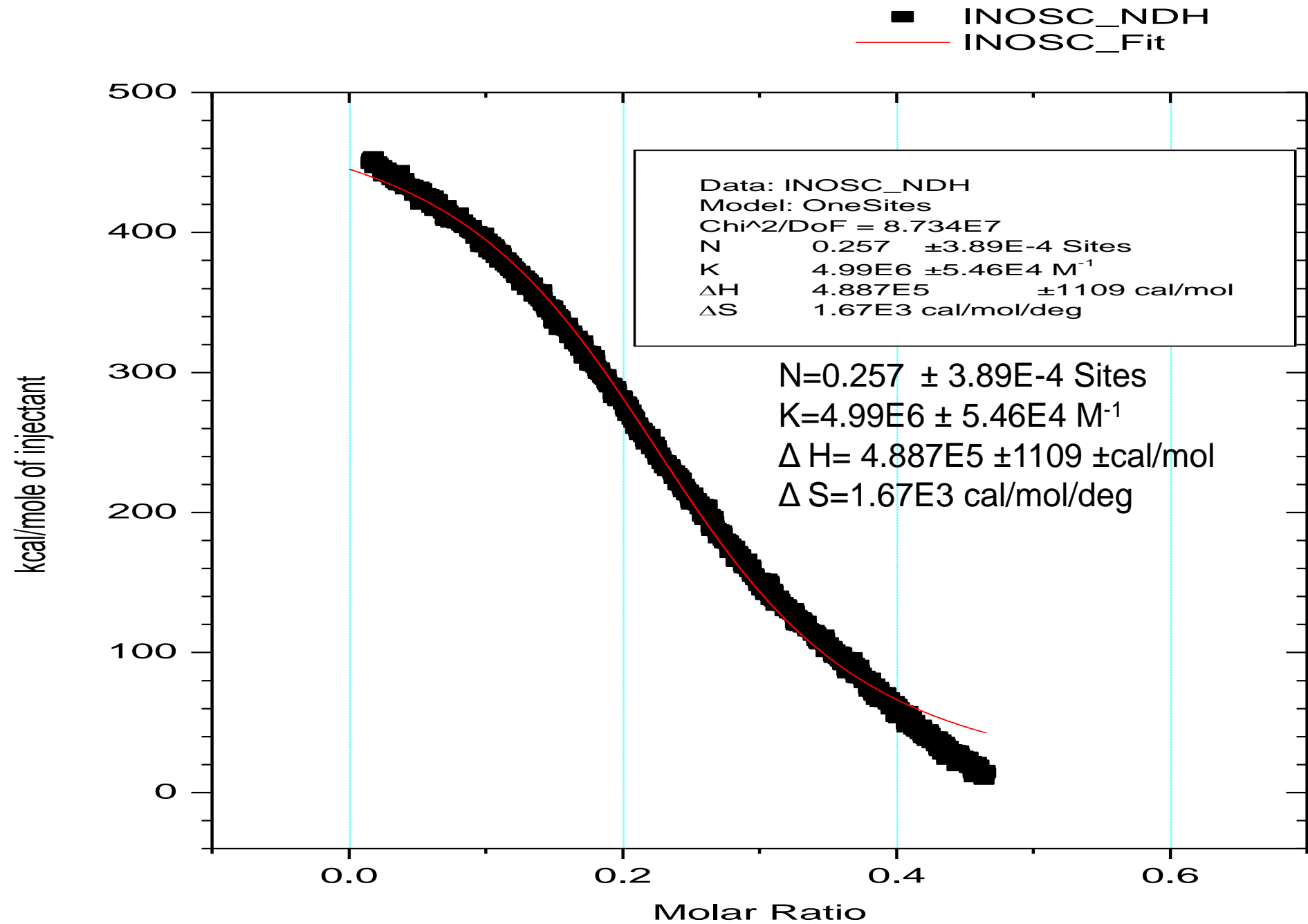

Supplement: S7 File — (PDF) [file pone.0240744.s007.pdf]
