# Supplementary material for: Correction: Characterization of Calmodulin-Free Murine Inducible Nitric-Oxide Synthase
Source: PLoS One. 2020 Oct 9;15(10):e0240744. doi: 10.1371/journal.pone.0240744 (PMC7546496; doi:10.1371/journal.pone.0240744)

1. **Relative Heme for iNOSfl proteins expressed and purified in the presence and absence of CaM.** UV-visible wavelength scans were recorded at room temperature on a Shimadzu UV-2450 spectrophotometer. For recording the spectra of CO binding to NOS, CO was first bubbled into the protein and sodium dithionite was further added and the proteins were subjected to wavelength scanning between 300 and 700 nm. The ferrous–CO adduct absorbance at 444 nm was used to determine heme content in the protein using an extinction coefficient of 74 $mM^{-1}\ cm^{-1}$ (A_{444}–A_{500})

Representative Heme-CO Spectra for iNOSfl purified in the presence and absence of CaM

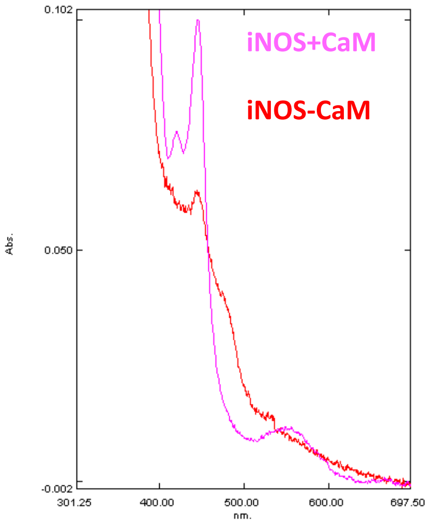

Heme content = [(O.D_{444}- OD_{555})] /74 * 1000 * dilution factor

2. **Steady state NO synthesis for iNOSfl proteins expressed and purified in the presence and absence of CaM -** Steady-state rates of NO synthesis activities were determined by the spectrophotometric oxyhemoglobin assay using a difference extinction coefficient of 38 $mM^{-1}\ cm^{-1}$ for the oxyhemoglobin to methemoglobin transition at 401 nm. The assay buffer used was the same as that of the Griess assay with addition of 10 µM oxyhemoglobin and 0.5 µM NOS was used for the assay. The reactions were initiated by addition of 10 mM NADPH and the absorbance change at 401 nm was recorded at 25ºC to quantify the amount of NO produced. Raw data value files for NO synthesis Assay by Oxyhemoglobin method are attached a Attachment#17B (iNOS+CaM) and Attachment#17C(iNOS-CaM)

Measurement of apparent K_m values for L-Arg or H_4B for iNOSfl proteins expressed and purified in the presence and absence of CaM

Apparent K_m values were determined by fitting plots of the NO synthesis activity *versus* L-Arg or H_4B concentration. In the case of K_m measurements for L-Arg, iNOSfl proteins were purified in the presence of L-Arg and H_4B and thereafter dialysed against a buffer with a higher concentration of H_4B (200 µM) and no L-Arg to remove L-Arg from the protein and obtain a H_4B bound iNOSfl protein. Corresponding NO synthesis activities of the protein was determined using different concentration of L-Arg (1–20 mM) and plotted to determine K_m of L-Arg for iNOSfl. Similarly, for the K_m measurements for H_4B, iNOSfl protein initially purified in the presence of L-Arg and H_4B was dialysed against a buffer with a higher concentration of L-Arg (20 mM) and no H_4B to obtain a H_4B-free, L-Arg bound iNOSfl protein. Corresponding NO synthesis activities were plotted similarly against different concentrations of H_4B (5–100 µM) to determine K_m of H_4B for iNOSfl

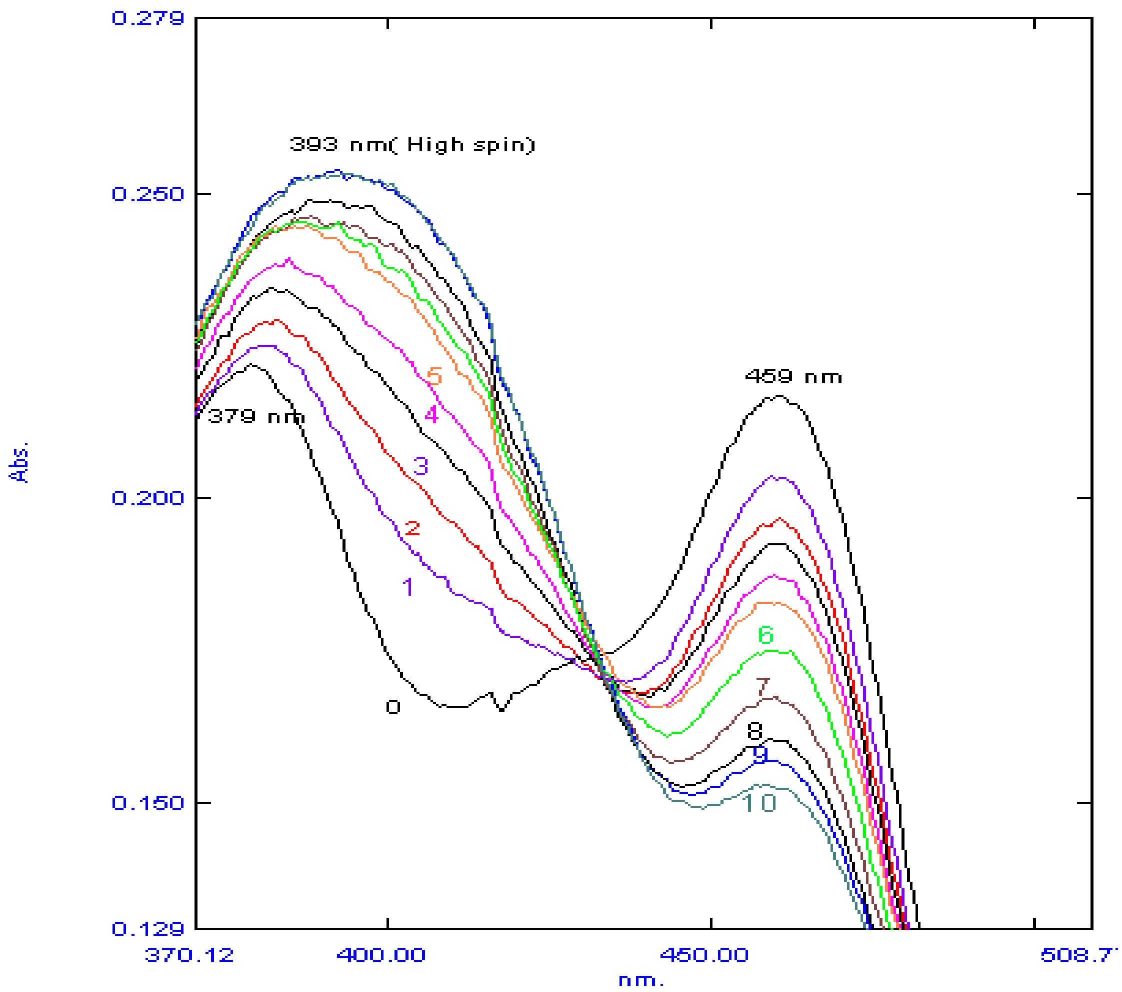

Spectra showing the gradual absorbance shift on addition of different concentration of tetrahydrobiopterin (H_4B)

Double reciprocal plot of absorbance difference at 393 nm vs H_4B concentration (μM)

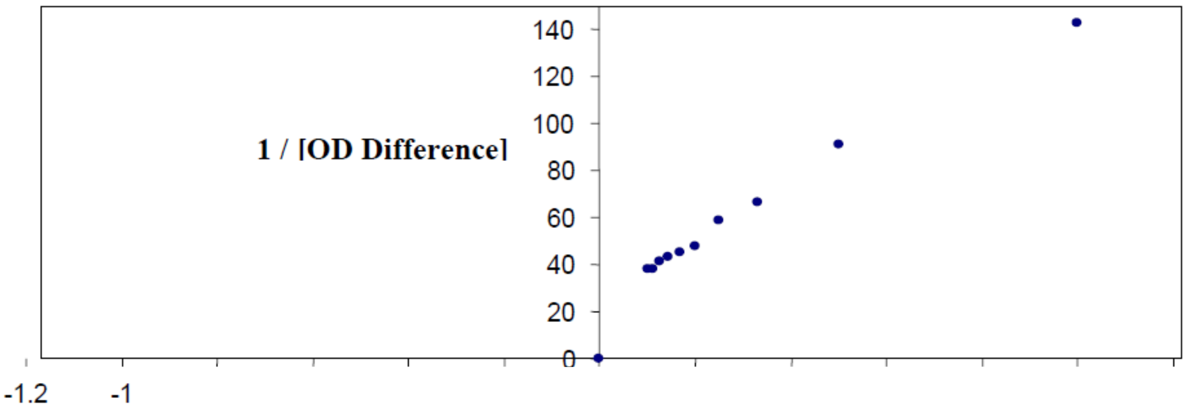

Supplement: S8 File — (ZIP) [file pone.0240744.s008.zip › S8 File/Attachment#17A_Table_1_Raw Data.pdf]
