# Supplementary material for: Correction: Characterization of Calmodulin-Free Murine Inducible Nitric-Oxide Synthase
Source: PLoS One. 2020 Oct 9;15(10):e0240744. doi: 10.1371/journal.pone.0240744 (PMC7546496; doi:10.1371/journal.pone.0240744)
Supplement: S9 File — (ZIP) [file pone.0240744.s009.zip › S9 File/Attachment#18A_Table_2_Raw Data.pdf]

1. **Ferricyanide reductase activity** - Ferricyanide reductase activities was determined by monitoring the change of absorbance at 420 nm, using extinction coefficients of 1.01 $mM^{-1}cm^{-1}$. The assays were spectrophotometrically carried out at 25°C.

The assay buffer contained 40 mM EPPS, pH 7.6, 4 µM FAD, 0.1 mg/ml BSA,10 µg/ml CaM, 0.6 mM EDTA, 10 units /ml catalase, 10 units/ml superoxide dismutase to which either 0.1 mM cytochrome c or potassium ferricyanide was added. After the addition of the iNOSfl enzyme (50nM), the reaction was initiated by adding 0.1 mM NADPH and the rate of reduction of cytochrome c and ferricyanide determined from the slopes of the reaction curves.

Representative Ferricyanide Reduction Curve for iNOSfl purified in the absence of CaM

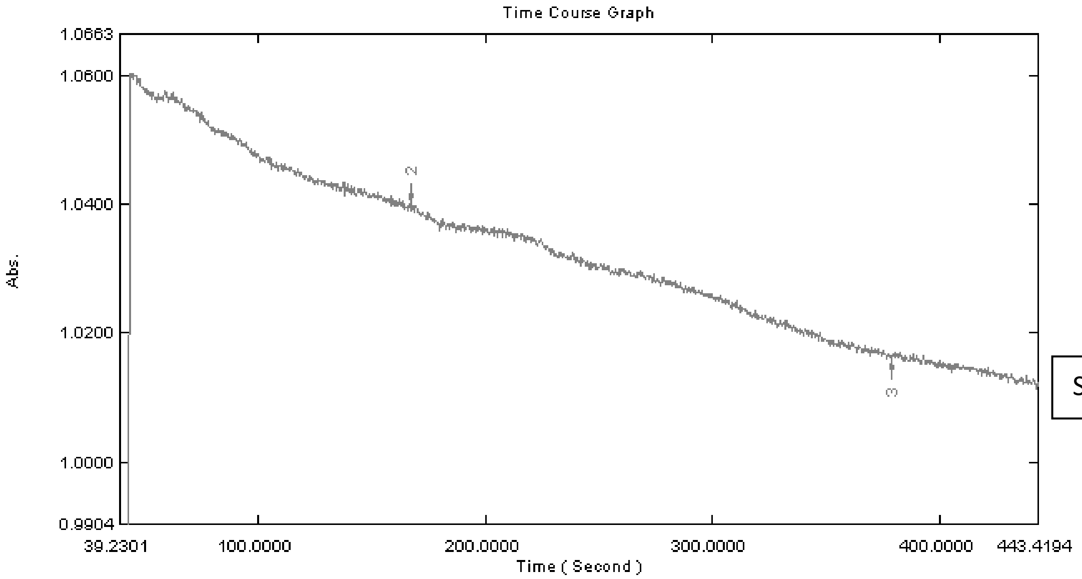

Slope-0.056

Representative Fericyanide Reduction Curve for iNOSfl purified in the presence of CaM

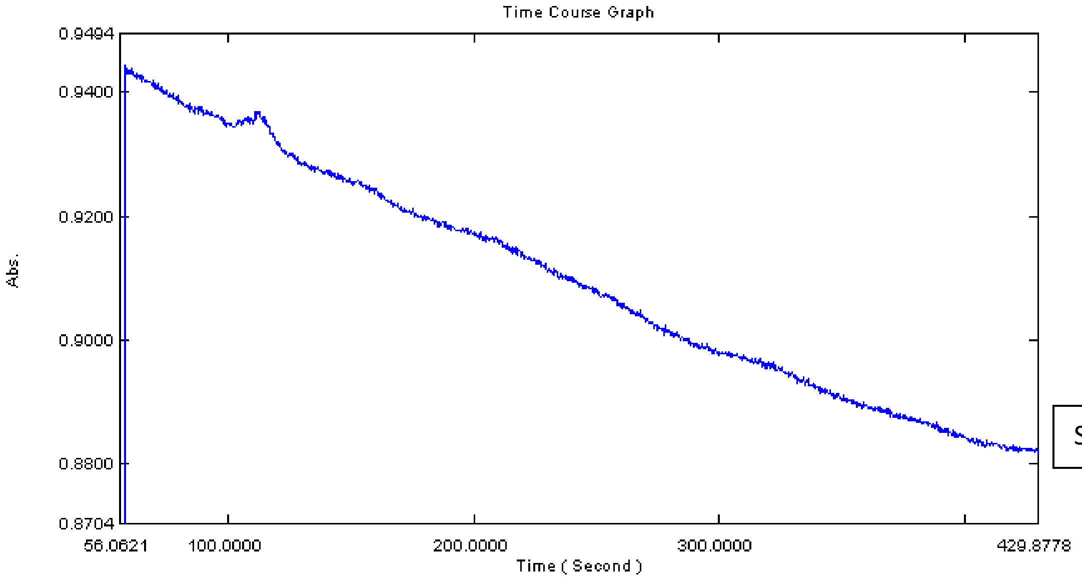

Slope-0.061

2. **Cytochrome c Reductase Activity**- The cytochrome c reductase activity was determined by monitoring the change of absorbance due to cytochrome c reduction by the iNOSfl protein at 550 nm using an extinction coefficient of 21 mM^{-1} cm^{-1}.

<u>Representative Cytochrome C Reduction Origin Plots for iNOSfl purified in the presence & absence of CaM</u>

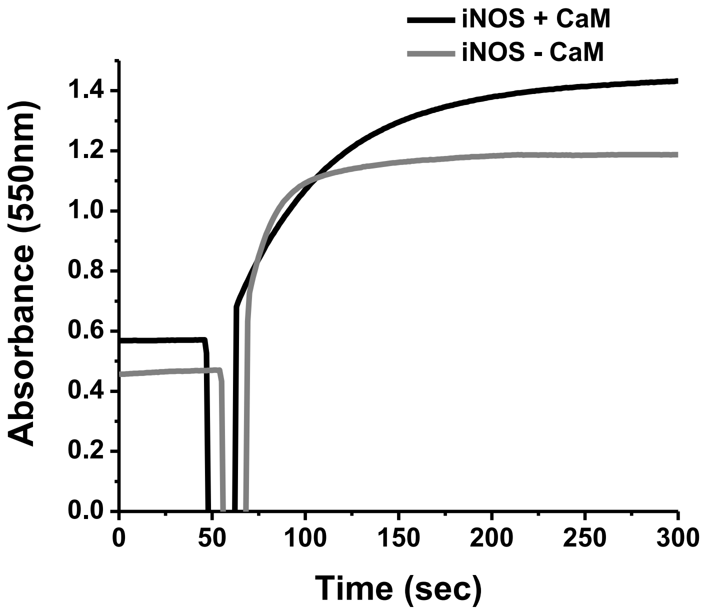

3. **Measurement of flavin content** - To calculate the total flavin content in iNOS proteins, flavin fluorescence emission spectra were measured by exciting samples at 450 nm and flavin fluorescence emission was measured from 470 to 650 nm. Total flavin was calculated using a flavin (FMN and FAD) standard curve with the same measurement parameters. All spectra were corrected for instrumental artifacts by subtracting the baseline emission spectrum of the buffer.

   Refer to Raw data text files Attachment#18B (iNOS+CaM) & Attachment#18C (iNOS-CaM) **for evaluation of Flavin content.**
